# A reciprocal feedback between the PDZ binding kinase and androgen receptor drives prostate cancer

**DOI:** 10.1038/s41388-018-0501-z

**Published:** 2018-09-20

**Authors:** Anne Y. Warren, Charlie E. Massie, Kate Watt, Katarina Luko, Folake Orafidiya, Luke A. Selth, Hisham Mohammed, Brinder S. Chohan, Suraj Menon, Ajoeb Baridi, Wanfeng Zhao, Carles Escriu, Thanakorn Pungsrinont, Clive D’Santos, Xiaoping Yang, Chris Taylor, Arham Qureshi, Vincent R. Zecchini, Greg L. Shaw, Scott M. Dehm, Ian G. Mills, Jason S. Carroll, Wayne D. Tilley, Iain J. McEwan, Aria Baniahmad, David E. Neal, Mohammad Asim

**Affiliations:** 1 Department of Pathology, Addenbrooke’s Cambridge University Hospital, Cambridge, UK; 2 Early Detection Programme, Cancer Research UK Cambridge Centre, Department of Oncology, University of Cambridge, Cambridge, UK; 3 Institute of Medical Sciences, University of Aberdeen, Aberdeen, UK; 4 Institute of Human Genetics, Jena University Hospital, Jena, Germany; 5 Dame Roma Mitchell Cancer Research Laboratories, School of Medicine, Faculty of Health Sciences, University of Adelaide, Adelaide, Australia; 6 Freemasons Foundation Centre for Men’s Health, School of Medicine, Faculty of Health Sciences, University of Adelaide, Adelaide, Australia; 7 Cancer Early Detection Advanced Research Center, Knight Cancer Institute, OHSU, Portland, USA; 8 Cancer Research UK Cambridge Institute, University of Cambridge, Cambridge, UK; 9 Masonic Cancer Center, University of Minnesota, Minneapolis, USA; 10 Prostate Cancer UK/Movember Centre of Excellence, CCRCB, Queens University, Belfast, UK; 11 Nuffield Department of Surgical Sciences, University of Oxford, Oxford, UK; 12 Department of Clinical & Experimental Medicine, Faculty of Health & Medical Sciences, University of Surrey, Guildford, UK

**Keywords:** Proteomics, Prostate cancer

## Abstract

Elucidation of mechanisms underlying the increased androgen receptor (AR) activity and subsequent development of aggressive prostate cancer (PrCa) is pivotal in developing new therapies. Using a systems biology approach, we interrogated the AR-regulated proteome and identified PDZ binding kinase (PBK) as a novel AR-regulated protein that regulates full-length AR and AR variants (ARVs) activity in PrCa. PBK overexpression in aggressive PrCa is associated with early biochemical relapse and poor clinical outcome. In addition to its carboxy terminus ligand-binding domain, PBK directly interacts with the amino terminus transactivation domain of the AR to stabilise it thereby leading to increased AR protein expression observed in PrCa. Transcriptome sequencing revealed that PBK is a mediator of global AR signalling with key roles in regulating tumour invasion and metastasis. PBK inhibition decreased growth of PrCa cell lines and clinical specimen cultured ex vivo. We uncovered a novel interplay between AR and PBK that results in increased AR and ARVs expression that executes AR-mediated growth and progression of PrCa, with implications for the development of PBK inhibitors for the treatment of aggressive PrCa.

## Introduction

Prostate cancer (PrCa) is a major cancer responsible for male fatalities worldwide [1]. The growth and survival of PrCa is driven by the male sex hormone androgens that act by binding to the androgen receptor (AR) [2, 3]. Consequently, androgen-deprivation therapy (ADT) is used as a first-line therapy for the treatment of locally advanced or metastatic PrCa. ADT comprises; luteinizing hormone-releasing hormone (LHRH) analogues that block androgen production, anti-androgens such as bicalutamide and enzalutamide that antagonise androgen binding to the AR, and abiraterone that inhibits the androgen biosynthesis pathway [4, 5].

ADT is initially effective but resistance is common and this leads to the development of aggressive incurable disease known as castration-resistant prostate cancer (CRPC) [6]. CRPC is often driven by the reactivation of AR signalling through multiple molecular mechanisms, which include amplification and gain-of-function mutations of the *AR* gene, gain-of-function mutations in the androgen signalling pathway [7], and the emergence of constitutively active AR splice forms known as AR variants (ARVs; e.g., ARV7). ARVs are devoid of the ligand-binding domain (LBD) and thus such variants cannot be targeted by ADT [8, 9]. Notably, the AR amino terminus domain (NTD) is the prime driver of PrCa growth, thus making it a potential drug target [10, 11].

In addition to direct changes to the AR, another mechanism underlying CRPC growth is increased activity and/or expression of AR co-activators [12–14]. Yet another hallmark of AR signalling in PrCa is the integrated feed-forward and feedback circuits that facilitate pro-growth signalling as exemplified by the reciprocal positive feedback between AR and choline kinase alpha signalling [14, 15] and reciprocal negative feedback between AR and poly ADP-Ribose polymerase-1 signalling [16]. Androgens can also negatively regulate AR mRNA expression by recruitment of AR to a repressive element in intron 2 of the *AR* gene [17, 18]. Despite decreasing AR mRNA levels, androgens promote AR stabilisation leading to overall increase in AR protein expression. This example highlights the critical need to analyse the androgen-regulated proteome in order to characterise androgen-regulated changes at protein level in PrCa.

In order to understand how AR is stabilised and promotes PrCa growth, it is imperative to identify clinically relevant AR targets, both up- and downstream of AR signalling. Here, we generated androgen-regulated proteome data sets to comprehensively identify effectors of androgen signalling and identified PDZ binding kinase (PBK) as an AR-regulated factor in vitro and in vivo in PrCa that integrates AR signalling with PrCa growth. In men with PrCa, PBK is androgen-regulated and localised in the tumour cell nucleus. PBK overexpression is associated with poor disease outcome and clinical progression. We further uncover PBK interactome and identify a crucial reciprocal feedback between PBK and AR, whereby PBK interacts with both the NTD and LBD of AR to directly regulate the stability and function of both full-length AR and ARVs. Inhibition of PBK activity destabilises AR and decreases PrCa growth and metastasis. These results provide novel insights into the role of androgen-induced pathways in PrCa revealing PBK as a key effector through which AR manifest its oncogenic function in PrCa.

## Results

### Identification of PBK as a novel AR target in PrCa

**Fig. 1:**
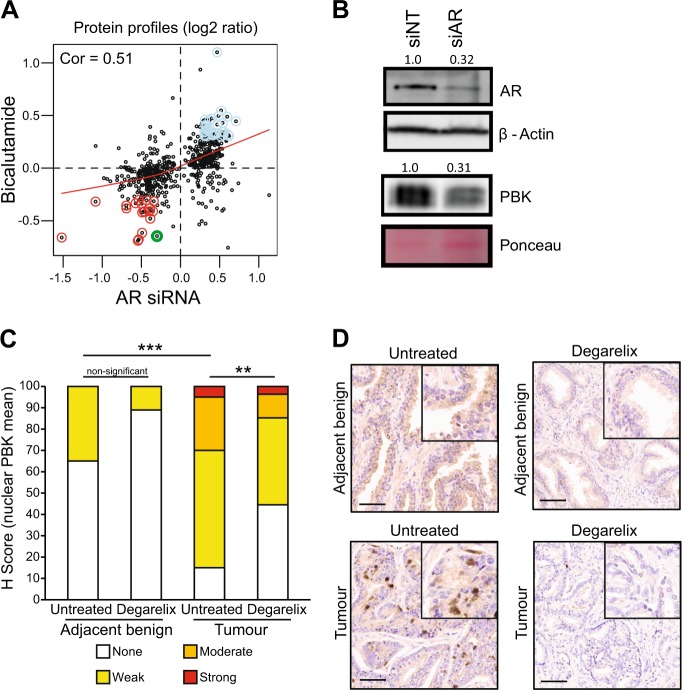
Identification of androgen-stabilised proteome in prostate cancer (PrCa). **a** Scatterplot showing the relationship between protein levels following AR knockdown and AR antagonist treatment of C4-2 cells grown in full media. Red line shows lowess regression, red points show commonly downregulated proteins, light blue points show commonly upregulated proteins (cor = 0.51). PBK is shown in green. **b** Western blot analysis showing of expression of PBK and AR proteins. C4-2 cells were transiently transfected with small interfering (si)-RNA targeting AR (siAR) or with non-targeting control siRNA (siNT); β-actin is the loading control for AR and the Ponceau for PBK. **c** Immunohistochemical staining score (H score) of nuclear PBK protein expression in tumours (in triplicates) and untreated benign adjacent epithelia (in duplicates) in patients with (*n* = 27) or without (*n* = 20) 7 days’ treatment with the LHRH analogue degarelix, statistical significance calculated by Mann–Whitney test. **d** Representative IHC images of corresponding tissue samples from degarelix-treated and untreated patients. Scale bars = 50 µm. H score ranged from 0 to 9 and converted it to a 4-point scale as 0 = none, 1–3 = weak; 4–6 = moderate and 7–9 = strong

Mass spectrometry identified the AR-regulated proteome of the CRPC cells following AR knockdown using a pre-validated set of four different siRNAs as used earlier [14] or AR inhibition with the AR antagonist, bicalutamide. Supporting the robustness of the data, a significant correlation was found between proteins regulated by AR knockdown and bicalutamide treatment (Pearson *r*^2^ = 0.51). The protein levels of known androgen-upregulated targets were downregulated by both AR knockdown and bicalutamide treatment, whereas androgen-repressed proteins were upregulated by both AR treatments (*n* = 514) (Fig. 1a and Table S1).

**Fig. 2:**
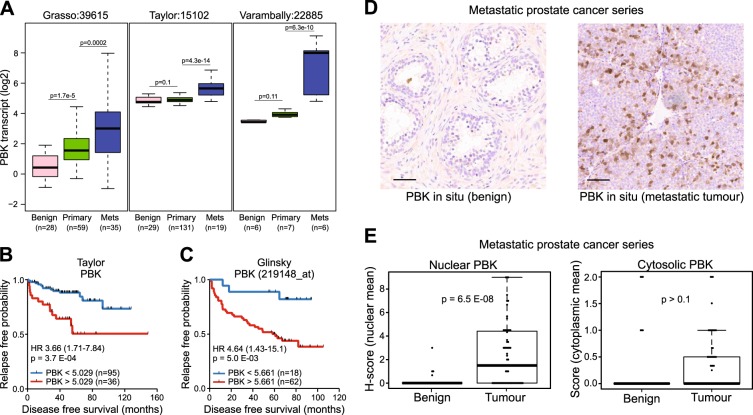
Clinical relevance of PDZ binding kinase (PBK) in PrCa. **a** Boxplots show PBK transcript expression in prostate tissues in three independent gene expression data sets [20–22]. Gene expression values (log2) of PBK trancript are shown. **b**–**c** Kaplan–Meier survival curve from recursive partitioning analysis. High levels of PBK transcript levels are associated with poor recurrence-free survival in the **b** Taylor cohort [21] and **c** in Glinsky data set [23]. **d** Representative IHC images of prostate samples from benign prostate glands and hormone refractory (metastatic) tumour tissue. Scale bars = 50 µm. **e**–**f** Corresponding Boxplot showing **e** nuclear and **f** cytosolic PBK protein levels in metastatic tumours from PrCa patients (*n* = 50), and benign specimen (*n* = 50) (*p* < 6.46e-06; Wilcoxon test for IHC)

A core set of 77 AR-regulated proteins common between AR knockdown and bicalutamide treatment was identified that showed consistent changes in the same direction, including known AR-regulated targets such as FKBP5 and FASN (Figure S1A and Table S2). This analysis also provided evidence that the vesicle transport protein NIPSNAP3A is an AR-repressed target. Among the factors in the proteome data that were commonly downregulated by both AR knockdown and bicalutamide treatment was PBK (Fig. 1a and Figure S1A). Supporting these findings, PBK transcript and protein levels decreased following AR knockdown (Fig. 2b, Figures S1B and S5A) and AR-binding sites were identified at the enhancer upstream of the *PBK* gene (Figure S1C). PBK expression in situ was also androgen-regulated in a series [19] of human prostate tissue comprising benign prostate tissues and PrCa samples from untreated men (*n* = 20) or those treated with androgen inhibitor degarelix (a LHRH agonist that blocks testicular androgen synthesis) for 7 days (*n* = 27) (Fig. 1c). Nuclear PBK staining was significantly higher in PrCa tissues compared with benign control tissues from untreated men ((*p* < 0.0001) (Fig. 1c, d). Degarelix treatment decreased the nuclear levels of PBK in the treated tumours compared with untreated patients (*p* < 0.01), with 45% of degarelix-treated cases having no detectable expression of PBK in comparison to 15% of untreated cases. Overall, cytosolic PBK levels were lower compared to nuclear levels in all tissues tested, whereas there was no significant difference in cytosolic PBK levels between tumours or benign tissue or in response to degarelix treatment (Figures S1D, S1E).

Taken together, these novel in vitro and in vivo data reveal that PBK is an AR-regulated factor whose expression is elevated in PrCa by androgen signalling. Given this knowledge and the amenability of kinases as potential drug targets, the role of PBK was further investigated in disease relevant models of PrCa.

### PBK overexpression identifies men with aggressive PrCa

Given the increased nuclear PBK expression in high-risk PrCa compared with benign prostate tissues (Fig. 1c), expression of PBK was examined in three independent clinical cohorts of men comprising benign, primary and metastatic PrCa [20–22]. In metastatic PrCa, tissue PBK transcript was upregulated in all three clinical cohorts when compared with benign and primary tumours (*p* = 0.0002, Grasso; [20] *p* = 4.3e-14, Taylor; [21] *p* = 6.3e-10, Varambally [22]) (Fig. 2a). The causative role of PBK in aggressive disease was supported by the recurrence-free survival analyses of two independent cohorts of clinical samples [21, 23], where PrCa patients with higher PBK levels experienced early biochemical recurrence (*p* = 3.2e-05 and *p* = 0.05) (Fig. 2b, c).

To further investigate the role of PBK in context of aggressive PrCa, in situ PBK expression was evaluated in a series of adjacent benign (*n* = 50) and metastatic PrCa (*n* = 50) tissues. Whereas PBK expression was low in benign tissue, metastatic tissue had significant higher PBK expression, which predominantly localised in the nucleus of tumour cells (*p* = 6.4e-08) (Fig. 2d, e) consistent with increased nuclear localisation observed in high-risk primary PrCa (Fig. 1d). No difference was found in the levels of cytosolic PBK in benign or metastatic PrCa epithelia. Together, these results indicate that PBK is overexpressed in aggressive PrCa and associates with biochemical recurrence. PBK is primarily nuclear in tumours, where its expression is restricted to epithelia and is significantly enhanced in CRPC compared with matched benign prostate tissue.

### PBK is an AR-stabilising factor that regulates AR expression

**Fig. 3:**
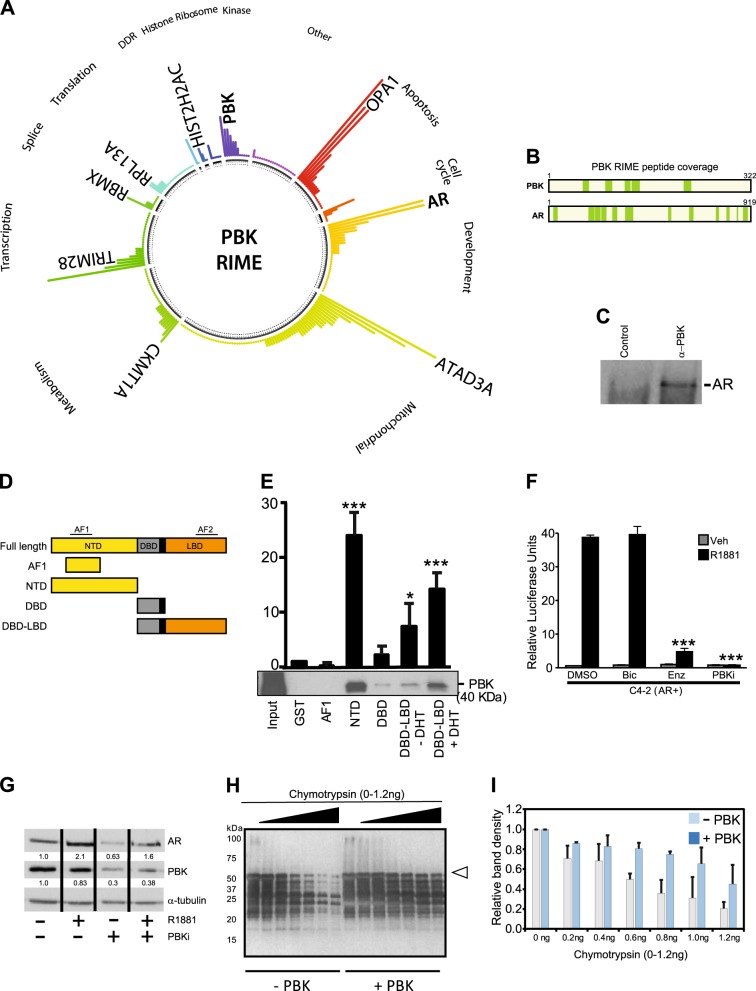
Interaction of PBK with AR and its effect on AR stability. **a** PBK RIME summary plot showing gene ontology categories (DAVID GO analysis) and key PBK-associated proteins identified in this experiment (filtered using IgG RIME) in C4-2 cells. Bar heights represent the unique peptide counts for each protein identified and the figure was drawn using the ‘circlize’ library in the R statistical package. **b** Peptide coverage of the PBK and AR proteins following PBK RIME assay in C4-2 cells. The locations of the identified peptides are highlighted in green blocks. **c** Co-immunoprecipitation showing interaction of endogenous AR with PBK. C4-2 Cell lysate were incubated with the PBK antibody followed by Western blot analysis to detect AR protein. A representative blot is shown (*n* = 3). **d–e** GST pull-down assay; **d** Schematic representation of AR domains used in pull-down. **e** GST alone and GST-tagged AR domains were incubated with recombinant PBK for 2 h and subjected to a pull-down assay. PBK was detected by immunoblotting with an anti-PBK antibody. The AR-DBD-LBD was expressed, purified and incubated ± 50 μM DHT. Fold interaction of each domain with PBK based on the intensity of the band as calculated by Image J software, GST = 1. A representative blot is shown (*n* = 2–5). **f** Luciferase reporter assay. AR transactivation potential in C4-2 cells transiently transfected with MMTV-luciferase plasmid and treated with androgen (1 nM R1881), bicalutamide, enzalutamide or PBKi (all 10 µM); (*p* < 0.001) bars show mean ± SD (*n* = 3). *p* values for two-sided Student’s *t* test. *** = *p* < 0.001. **g** Western blot showing protein expression of PBK and AR in C4-2 cells. Cells were treated with R1881 (1 nM) or PBKi (1 µM) for 48 h; Tubulin is the loading control. A representative blot is shown (*n* = 3). **h** Representative Western blot for AR^NTD^ digested with chymotrypsin in the absence or presence of recombinant PBK. Open triangle represents the full-length AR^NTD^. Proteolysis was performed for 5 min in the presence of increasing concentrations of chymotrypsin (0–1.2 ng). AR^NTD^ fragments were detected by Western blot using the anti-androgen receptor antibody ab3510 (Abcam) corresponding to human AR amino acids 1–21 (N-terminal). **i** Quantitation of the AR^NTD^ full-length polypeptide, after digestion with chymotrypsin for two independent experiments. PBK = PDZ binding kinase; AR = androgen receptor; IP = immunoprecipitation; AF = activation function; NTD = amino terminal domain; DBD = DNA binding domain; LBD = ligand-binding domain; GST = glutathione-s-transferase; DHT = dihydrotestosterone, DMSO = dimethylsulphoxide

PBK is known to be an important regulator of cellular processes such as transcription and mitosis [24–26], but its input in PrCa is illusive. To understand the role of PBK in PrCa, we employed rapid immunoprecipitation mass spectrometry (RIME)-based targeted proteomics approach [27] to identify interaction partners of PBK in PrCa globally. We hypothesised that identifying such interacting factors could provide insight into the function of PBK in PrCa. RIME assay identified multiple unique PBK peptides, validating the target enrichment and experimental robustness in C4-2 cells (Fig. 3a, b). Proteins co-precipitating with PBK included mitochondrial factors (ATD3A, ATD3B, ATPB, COX2, COX41, SCO1, SCO2, SDHA), members of the histone protein family (H2A1A, H2A2C), which are known to be phosphorylated by PBK [28], a number of proteins involved in cancer metabolic pathways (ACSL3, ADT2, ADT3, PGK1) and heat-shock protein family members (HSP76, HSPB1) (Table S3).

Interestingly, the PBK interactome also identified AR, providing potential evidence for a cross-talk between the androgen and PBK signalling pathways and supported by the co-immunoprecipitation (Co-IP) experiments further validating the interaction between PBK and AR (Fig. 3c and Figure S5B). To map the domain(s) of AR that interact with PBK, we used GST pull-down assays (Fig. 3d), which showed that PBK physically interacts with both the amino terminus (AR^NTD^) and the ligand-binding domain (AR^LBD^) of AR (Fig. 3e and Figure S5C).

Given their direct interaction, we tested the impact of PBK inhibition either by PBK knockdown using a pre-validated pool consisting of four different siRNAs targeting distinct regions on the PBK mRNA or its chemical inhibition using HI-TOPK32 compound (PBKi) [29] as a proof-of-principle. A significant knockdown of PBK was observed in C4-2 cells transfected with a pool of four different siRNA targeting the PBK transcript (siPBK ON-TARGET plus) (*p* = 0.000; over 90% decreased PBK transcript abundance) or treated with PBKi (*p* = 8.24e-06) (Figures S2A, S2B). PBK inhibition reduced androgen (R1881)-induced AR transcriptional activity approximately 40-fold (Fig. 3f). The extent of AR inhibition by PBKi was comparable to enzalutamide (*p* < 0.001) and significantly greater compared with bicalutamide.

PBK knockdown also decreased the androgen-induced transcriptional activity of full-length AR in the other CRPC model cell line CWR-R1-AD1 (*p* < 0.05) (Figure S2C). As we found the interaction of PBK with the NTD of AR in addition to its LBD, we performed reporter assay in R1-D567 cells, that harbour the ligand-independent ARv567es variant of AR that is exclusively activated by its NTD and lacks a functional LBD. As expected, neither of the LBD targeting anti-androgens bicalutamide or enzalutamide was able to inhibit transcriptional activity of ARv567es, however both PBK knockdown (*p* < 0.01) and PBKi (*p* < 0.01) decreased AR transcriptional activity (Figure S2D), indicating its superiority over conventional anti-androgens. PBK knockdown also decreased expression of two endogenous AR targets, TMPRSS2 and NKX3.1 in C4-2 cells (Figure S2E). PBK-mediated effects on AR transcriptional activity were not related to *AR* gene expression as neither PBKi nor PBK knockdown affected AR mRNA levels or AR promoter activity (Figures S2F, S2G). However, PBKi decreased both basal and androgen-stabilised AR protein levels in PrCa cells (Fig. 3g and Figures S5D, S5E), suggesting that PBK inhibition decreases AR protein expression by a post-translational mechanism. As AR is activated and stabilised by phosphorylation, we also tested the possibility of PBK-mediated AR phosphorylation by phosphoproteomics using PBKi but failed to identify differential phosphorylation events on AR (data not shown). To understand the mechanism by which PBK impacts AR protein expression, a protease protection assay using a recombinant AR^NTD^ polypeptide was performed. In this assay inclusion of a non-binding protein such as GST does not inhibit proteolysis of recombinant AR domains, whereas AR-binding factors significantly resist proteolysis [14]. In line with this, in the absence of recombinant PBK, AR^NTD^ polypeptide was sensitive to chymotrypsin-mediated degradation, but the presence of PBK antagonised chymotrypsin-mediated degradation of the AR^NTD^ and a subset of fragments, at both low and high concentrations of chymotrypsin, indicating that direct binding of PBK to AR^NTD^ confers AR stability (Fig. 3h, i). This protection of AR^NTD^ by PBK was not observed in the presence of trypsin, consistent with a discrete binding site (Figures S2H, S2I). Collectively, these experiments suggest that PBK directly interacts with the AR^NTD^ and AR^LBD^, resulting in its stabilisation and enhanced transcriptional activity in PrCa.

### PBK signalling regulates a distinct transcriptome in PrCa

To test whether PBK signalling affects the AR-regulated transcriptional programme in PrCa, we performed RNA sequencing (RNAseq) to test the global impact of PBK inhibition by either siPBK or PBKi. A significant overlap in the gene transcripts affected by PBK knockdown and PBKi was observed (false discovery rate; FDR < 0.05) for genes downregulated following PBK inhibition (hypergeometric *p* = 1.7e-9) (Figure S3A). Functional annotations for DNA damage repair, cell cycle and many other Cancer Hallmarks [30] pathways were enriched among the genes altered in response to PBK inhibition or PBK knockdown (FDR-adjusted *p* values shown; Figure S3B), consistent with functions previously attributed to PBK signalling [24, 28].

**Fig. 4:**
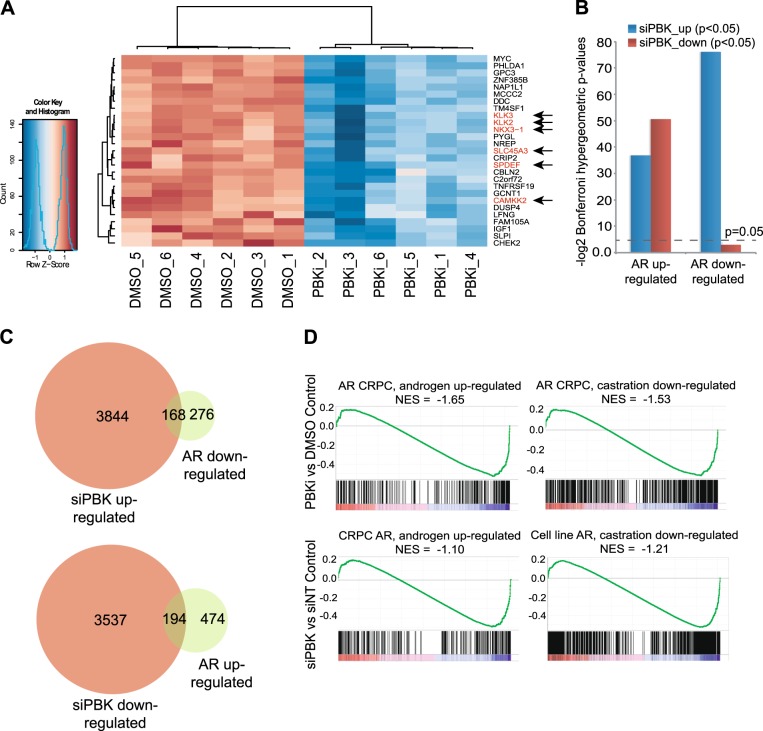
The PBK-regulated transcriptional programme in PrCa. **a** Heatmap showing differentially expressed genes from RNAseq analysis of PBK inhibitor (PBKi) and vehicle control (DMSO) treated C4-2 prostate cancer cells. Heatmap shows z-scores and known AR-regulated genes are highlighted in red and with arrows. **b** Barplot showing significance testing of the overlaps between PBK inhibitor or PBK knockdown and AR gene sets. Bonferroni corrected *p* values from hypergeometric tests are plotted as inverse log-values. **c** Venn diagrams showing the overlap between PBK and AR-regulated genes (overlaps used to calculate *p* values in **c**. **d** Gene set enrichment analysis (GSEA) plots showing the enrichment of AR gene sets in the PBK inhibitor and PBK knockdown ranked expression profiles

Many classic AR-regulated genes showed altered expression following PBK inhibition (KLK3/PSA, KLK2, NKX3-1, SLC45A3, SPDEF, CAMKK2; Fig. 4a) and we found enrichment of AR-regulated metabolic pathways [31] further highlighting the interplay between AR and PBK expression and activity. In line with PBK affecting AR stability, there was a significant overlap between PBK-regulated genes and known AR-regulated gene sets [31] as 37% of AR downregulated genes and 29% of AR upregulated genes were reciprocally regulated by PBK knockdown (Bonferroni corrected hypergeometric test *p* < 0.01; Fig. 4b, c). Next, we assessed the enrichment of AR gene sets in the PBK gene expression profile using gene set enrichment analysis [32], which revealed a directional, quantitative enrichment of AR gene expression signatures in both PBK knockdown and PBK inhibitor expression profiles (Fig. 4d). These focussed analyses strongly support PBK mediated regulation of AR transcriptional output in PrCa cells.

### PBK regulates PrCa growth and invasion

**Fig. 5:**
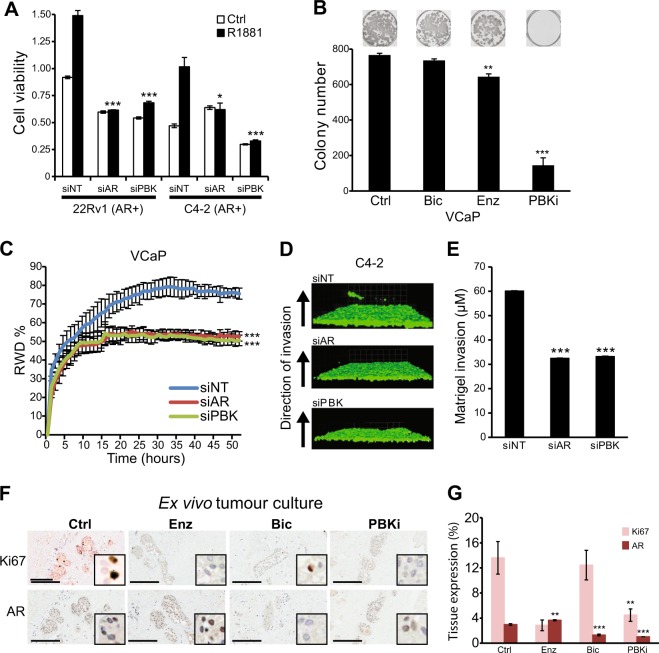
Regulation of PrCa growth and invasion by PBK signalling. **a** MTS cell viability assay of 22Rυ1 and C4-2 cells transfected with indicated siRNA targeting AR (siAR) or PBK (siPBK) or non-targeting control (siNT). Cells were grown in the presence or absence of R1881 for 5 days or near confluence; bars show mean ± SD (*n* = 3). *p* values for two-sided Student’s *t* test. **b** Clonogenic cell survival assay in VCaP cell lines treated with bicalutamide, enzalutamide or PBKi (14-day assay, all at 10 µM). **c** Scratch–wound assay. VCaP cells were transiently transfection with siNT, siAR or siPBK and wound closure was monitored, error bars represent ± SD (*n* = 4). Significance calculated by two-way ANOVA. **d**–**e** Matrigel-based inverted invasion assay **d** photomicrographs and **e** quantification of GFP tagged C4-2 cells transiently transfected with indicated siRNA; bars show mean ± SD (*n* = 3), significance calculated by D’Agostino & Pearson omnibus normality test *p* < 0.0001. **f** Photomicrographs of PrCa tissue explants treated with indicated drugs. Scale bars = 200 µm. **g** Barplot shows nuclear expression of AR and Ki67 in human PrCa tissue grown ex vivo and treated with bicalutamide, enzalutamide or PBKi (10 µM); bars show ± SEM (*n* ≥ 3). *p* values by two-sided Student’s *t* test. ** = *p* < 0.01; *** = *p* < 0.001. RWD = relative would density; Si = small interfering; NT = non-targeting; AR = androgen receptor; PBK = PDZ binding kinase; Ctrl = control; Bic = bicalutamide; Enz = Enzalutamide; PBKi = PDZ binding kinase inhibitor

To understand the functional consequence of the reciprocal feedback between AR and PBK on PrCa cell growth, cell growth assays were performed. In AR-positive cell line models of CRPC, such as 22Rυ1, C4-2 and LNCaP-Bic cells, PBK knockdown decreased androgen-induced cell growth (Fig. 5a and Figure S4A), however, the growth of AR-null PC3 or DU145 cell lines was marginally repressed by PBK knockdown (Figure S4B). These experiments indicated the requirement of PBK for AR-driven PrCa, which was further confirmed using clonogenic assays, in which PBK inhibition decreased clonogenic potential of AR-positive VCaP and DUCAP cell lines (Fig. 5b and Figure S4C).

As PBK expression was significantly upregulated in metastatic PrCa, and the PBK-regulated transcriptome showed pathways enriched for ‘cell migration/invasion’ (Figure S3B), hence we tested whether PBK activity conferred an invasive phenotype to PrCa cells. Both, PBK or AR knockdown decreased wound closure to a comparable extent (*p* < 0.001), indicating delayed migration of cells in scratch–wound assays (Fig. 5c). Concordantly, PBK knockdown or treatment with PBKi decreased PrCa cell invasion in a three-dimensional matrigel-based invasion model (*p* < 0.001) (Fig. 5d, e and Figures S4D, S4E). These findings were further validated in Boyden Chamber trans well migration assays (*p* < 0.001) (Figure S4F). Next, we assessed the relevance of PBK for PrCa growth and AR expression in a more clinically relevant model, in which hormone-naive primary patient tumours were treated in an ex vivo culture assay with PBKi. In this assay, both enzalutamide or PBKi, but not bicalutamide resulted in decreased tissue proliferation (~ 4 and ~ 10-fold, respectively; *p* < 0.01) as assessed by Ki67 staining. AR levels were reduced by bicalutamide and PBKi, in both cases to levels approximately threefold lower than the control treatment (*p* < 0.001) (Fig. 5f, g). These experiments provide robust experimental evidence for the key role of PBK in sustaining AR levels and tumour growth, indicating that PBK is an essential player in PrCa cell survival and invasion.

## Discussion

**Fig. 6:**
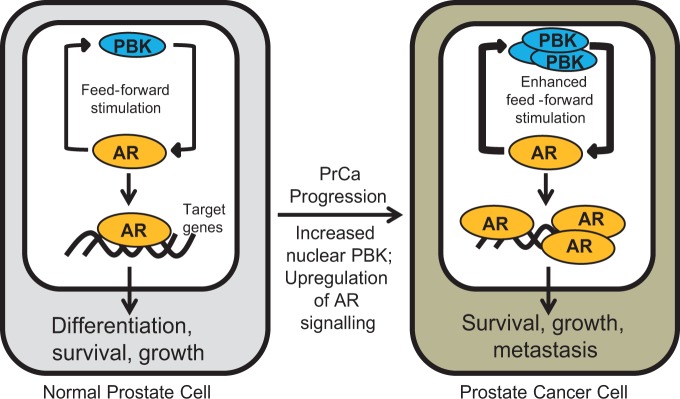
Model depicting oncogenic role of PBK in PrCa. In normal prostate epithelia, AR activity is optimally controlled by normal levels of PBK to allow cellular differentiation, survival and growth. PBK overexpression can result in the progression of PrCa by increasing AR activity leading to uncontrolled growth and metastasis of PrCa cells. PBK overexpression could also lead to AR increased AR stability and increased AR signalling allowing more PBK production hence hyperactivating the feed-forward stimulatory loop between AR and PBK. AR = androgen receptor; PBK = PDZ binding kinase

We performed mass spectrometry of PrCa cells to identify AR-regulated proteome, with a view to identifying novel effectors downstream of this key oncogene that promote PrCa progression in response to androgen signalling. Among these, we uncovered PBK as a novel AR-regulated protein whose expression is directly regulated by androgens in vitro and in men with PrCa. Interestingly, PBK expression was predominantly nuclear in PrCa and identified aggressive disease subtype suggesting its potential as a prognostic biomarker for cancer progression. Mechanistically PBK forms a feed-forward AR-PBK signalling loop whereby PBK binding to the NTD and LBD of the AR promotes its stability and activity. As a consequence, PBK overexpression observed in aggressive disease could potentially serve as a survival strategy in promoting AR stability, stimulating growth, migration and invasion of PrCa. These findings are summarised in Fig. 6.

PBK has emerged as a key player in multiple tumour types, including breast cancer [33], haematological malignancies [34], cervical cancer [35] and bladder carcinoma [36]. PBK appears to represent an important nexus for various signalling networks that promote cancer proliferation and invasion and thus *pbk* can exploit other oncogenic triggers to enhance its own expression. For example, the cytokine IL-6, which is overexpressed and promotes PrCa progression can upregulate PBK expression [37]. Likewise, oncogenic transcription factors such as ETS, c-Myc and E2F, which are all activated in PrCa, have been shown to drive PBK expression in various cancer types [38–40].

In advanced PrCa, AR is the major therapeutic target, and its point mutations such T878A and F876L, as well as the LBD deleted splice variants can lead to a constitutively active AR leading to failure of ADT. There is evidence that reducing AR protein levels may be more effective in treating CRPC than inhibiting its activity [14, 41]. As NTD is the major transactivation domain driving AR activity, it has recently emerged as a novel therapeutic target [42]; however, given the intrinsically disordered nature of the NTD, agents targeting NTD such as EPI-001 have shown to be non-specific [43]. As PBK can directly interact with the NTD of AR to oppose its degradation by proteases in a chaperone-like fashion, inhibition of PBK represents an attractive therapeutic strategy to degrade NTD of AR and thus inhibiting overall AR activity in PrCa. Indeed, PBK is not the first kinase that interacts with the AR. Previously, we and others have shown that a number of kinases can interact with the AR [14, 44–47], whereby their primary function appears to be the catalytic activation of the AR. In addition, we recently discovered that a kinase can also act as protein chaperone with the example of choline kinase alpha that can directly stabilise AR LBD independent of its catalytic function [14]. However, PBK exerts a unique effect on the AR as it is the first kinase to our knowledge that can interact with both LBD and NTD of AR and can directly stabilise the NTD of AR in a chaperone-like fashion. Overexpression of PBK in advanced PrCa appears to maintain the expression and activity of both full-length (wild-type and T878A mutant) AR and its splice variants, both of which are driven by NTD of AR. Given the stabilisation of AR protein levels by PBK, therapeutically relevant PBK-targeting agents would be predicted to degrade both full-length as well as splice variants of ARs, which are driven by NTD of AR.

Our findings suggest that AR promotes PrCa growth via upregulation of PBK and the therapeutic benefits of anti-androgens could result from inhibition of PBK in primary PrCa. We believe that the primary reason for the development of CRPC could be the loss of regulatory axis whereby, instead of AR, PBK expression is regulated by other oncogenic transcription factors such as E2F and c-myc [38, 40] as a result of inhibition of androgen signalling initially achieved by the anti-androgenic drugs. Upregulation of PBK in CRPC appears to serve an important strategy to stabilise AR expression and signalling and could provide an explanation for the enhanced AR activity, which is associated with poor prognosis of CRPC. In this context, our findings that PBK stabilises AR in advanced disease provide a mechanistic basis for increased AR activity and a rationale for therapeutic targeting of PBK for the treatment of CRPC. Thus, we propose that therapeutically targeting of PBK alone or its rationale combination with AR inhibitors is a novel and attractive treatment approach for the otherwise fatal CRPC. Small peptides-based medicinal chemistry approaches to synthesise PBK inhibitors that can disrupt the interaction between AR and PBK in CRPC will be ideally suited to develop precise targeting approaches and in order to effectively inhibit PBK-mediated AR activation and avoid off-targets effects often observed with kinase inhibitors-based therapy. In summary, our novel interrogation of the AR-regulated proteome has identified PBK as an novel AR-associated factor that can activate both LBD and NTD of AR and can lead to enhanced AR signalling that combined with its known key function in mitosis [26], represents a new therapeutic target in advanced PrCa.

## Materials and methods

### Reagents/consumables

Methyltrienolone was obtained from Perkin Elmer, enzalutamide was obtained from Axon Medchem and bicalutamide was obtained from Sigma chemicals. Dihydrotestosterone, dimethyl sulfoxide (DMSO) were obtained from Sigma chemicals (Dorset, UK). The PBK inhibitor HI-TOPK-032 (PBKi) was purchased from InterBioScreen, Russia. Life Technologies (Carlsbad, CA) provided cell culture media, fetal bovine serum (FBS), antibiotics etc.

### Cell lines

Genetic profiling of polymorphic short tandem repeat loci was used to verify cell lines in accordance with ATCC guidelines. AmpFISTR test or GenePrint10 test (Promega, Madison, WI) was used, and data were analysed using GeneMapper v4.0. LNCaP, C4-2, VCaP, PC3, DUCaP, 22Rυ1 and DU145 cells were procured through commercial suppliers. These cell lines were grown in Roswell Park Memorial Institute media that contained 10% FBS and 1% penicillin/streptomycin in a humidified incubator at 37 °C with an atmosphere of 5% CO_2_.

R1-AD1 is a CWR-R1 subline whose identity was verified using polymerase chain reaction (PCR) and Sanger sequencing (positive for the H874Y point mutation in the AR ligand-binding domain). Multiple ligation-dependent probe amplification assay was used to rule out copy number imbalances along the entire length of the *AR* gene. PCR and Sanger sequencing methods were also used to verify the identity of R1-D567 cell line to detect the transcription activator-like effector endonuclease-based genome engineering-induced signature break fusion junction.

### SILAC labelling/mass spectrometry

C4-2 cells were labelled with either with heavy or light amino acids, and transfected with ON-TARGET plus siRNA pool (and non-targeting control siNT) for 72 h or treated with anti-androgen Bicalutamide or DMSO (24 h). Cells were washed twice with cold PBS, lifted, and were centrifuged (300–400 g) at 4 ^o^C. Cells were mixed together (siNT heavy with siAR light or vice versa; DMSO heavy with bicalutamide light or vice versa). Supernatant was discarded and cells were lysed using lysis buffer (8 M urea, 50 mM ammonium bicarbonate, phosphatase, and a cocktail of protease inhibitors). Protein lysates (~30 µg in 8 M urea buffer) were loaded onto one-dimensional sodium dodecyl sulphate gel and run ~ 1.5 h to get a full-length gel with complete separation. For in-gel digestion, the gel was cut into 18 pieces, which were briefly washed with ammonium bicarbonate buffer, and further reduction was carried out with dithiothretol and alkylation with Iodoacetamide, which was followed by trypsin digestion (conc. 1:100, enzyme: substrate) at 37 °C overnight. 0.4% of trifluoroacetic acid was used to stop the digestion reaction and the supernatant was transferred to a reaction tube following by drying using speed vac. Peptides were re-dissolved in 6 µl of 2% acetonitrile/0.1% formic acid solution, and 5 µl was loaded with auto sampler (Ultimate3000 nano RSLC HPLC, Dionex, Thermo-Scientific, Waltham, MA) onto a trapping column (100 µm, 2 cm, 3 µm), separation was achieved with an Easy-spry Pep map analytical column (75 µm, 25 cm, 2 µm). Both were packed with C_18_ resin (100-Å pore size; Acclaim PepMap RSLC, Thermo-Scientific). Peptides were separated with an Ultimate3000 nano RSLC (Dionex) HPLC with in-line flow rates of 4.0 and 0.35 µl/min, respectively). The LC gradient range was from 5 to 35%B in 100 min (buffer A: 0.1%FA-5%DMSO; buffer B: 0.1%FA-75%MeCN-5% DMSO). The eluted peptides were analysed using the Q Exactive Hybrid Quadrupole-Orbitrap Mass Spectrometer (Thermo-Scientific). One full MS scan (at 400–1800 *m*/*z*; acquired at 70,000 resolution and automatic gain control (AGC) target of 10^6^) for each cycle was performed. This was followed by 10 data-dependent MS/MS spectra (AGC target, 5 × 10^4^, 17,500 resolution). Selected ions were dynamically excluded for 30 s and singly charged ions were rejected using charge-state screening.

### Database searching and data-filtering

The RAW files were processed with Thermo Proteome Discoverer 1.4. The MS/MS spectra were searched using both Sequest and Mascot against a reference composite database that contained the Human Uniprot protein database (downloaded April/2014 from http://www.uniprot.org/). Search parameters included partially tryptic specificity; fragment mass tolerance of 0.05 Da; a precursor mass tolerance of 20 ppm; a static modification of carboxy amidomethylation; and dynamic modifications of oxidation and de amidation. Results were filtered to only include fully tryptic peptides. Other cutoffs were established to achieve maximum sensitivity levels at < 0.1% false-positive rate (one reversed sequence hit for every 1000 forward sequence hits).

### RIME assay

This was performed on C4-2 cells using a PBK antibody (Novus) following a recently developed RIME method [27].

### Plasmids, siRNA and transient transfections

All plasmids were sequence verified. Four different and highly target specific siRNA sequences (ON-TARGET plus smart pool of four-independent siRNAs) were used to achieve potent knockdown of AR and PBK (Life technologies, Carlsbad, CA). Lipofectamine RNAiMAX transfection reagent (Life Technologies) was used to perform transient transfections with siRNA. Lipofectamine2000 transfection reagent (Life Technologies) was used to perform DNA transfection following manufacturer’s protocols. Reverse transfections were performed using 25 nM siRNA in all knockdown experiments except for RNAseq, where 50 nM siRNA was used to attain a near complete depletion of endogenous PBK. MMTV-Luc and AR-Luc reporter plasmid have described earlier [14, 48].

### Reporter assays

Cells treated with androgens were grown in medium containing hormone-depleted (charcoal-stripped) FBS. Renilla luciferase (Promega, Madison, WI) was used as an internal control in all luciferase assays. Following 48 h transfection, cells were harvested in passive lysis buffer (Promega) and then luciferase assay buffer was used to measure both luciferase/Renilla luciferase activity.

### Real-time qPCR

Cells were transiently transfected using RNAiMAX reagent with siRNA (25 nM) for 48 h. Cells were then grown in androgen-deprived charcoal-treated RPMI medium and treated with R1881 (1 nM) for 24 h. High Capacity cDNA Reverse Transcription Kit (Applied Biosystems, Carlsbad, CA) was form for cDNA preparation. For PCR, we used Taqman quantitative real-time probes (Applied Biosystems) to quantify gene expression changes in TMPRSS2 and NKX3.1 relative to 18 S expression, which served as the internal control. AR knockdown was used as the positive control; experiments were carried out with nine replicates.

### Cell viability and clonogenic assays

To determine cell viability, cells were incubated with 3-(4,5-dimethylthiazol-2-yl)-5-(3-carboxymethoxyphenyl)-2-(4-sulfophenyl)-2H-tetrazolium) reagent. This reaction was followed by colorimetric assay using Promega’s cell viability kit protocol. Clonogenic assays were performed using the exact methodology, which has been described earlier [14].

### PBK binding to the AR-NTD: stabilisation of receptor polypeptides

AR domains were purified as described previously [14]. In total, 25 pmoles of AR-NTD recombinant protein was treated with chymotrypsin in the presence and absence of PBK (25 pmoles). Proteolysis with chymotrypsin was performed for 5 min in the presence of increasing concentrations of chymotrypsin (0–1.2 ng). Trypsin digestion was performed at a constant concentration of 1 ng with an increase in time from 0 to 20 min. AR-NTD fragments were detected on western blots using the anti-AR antibody ab3510 (Abcam) corresponding to Human AR amino acids 1–21 (N-terminal).

### GST pull-down assay and Co-IP

These experiments were performed as described earlier [14]. For GST pull-down assay, 50 pmoles of PBK (ab123196, Abcam) antibody was used for immunoprecipitation and anti-PBK (ABnova Corp. Taiwan; 1:2500 dilution) and goat-antimouse HRP antibody was used for western blotting. For Co-Ip experiments anti-PBK Abcam dilution 1/30; anti-AR Santa Cruz N-20 dilution 1/30 were used. Statistical significance was calculated using two-tailed Student’s *t* test when *n* = 3 or more.

### RNAseq and comparison with the AR transcriptome

C4-2 cells were treated with PBKi for 6 h or transfected with PBK siRNA for 72 h. Qigen Allprep kit (Qiagen, Hilden, Germany) was used to isolate total RNA, which was quantified using Qubit (Life technologies) and quality (RIN > 8) was confirmed using the Bioanalyzer 2100 (Agilent). TruSeq® mRNA HT Sample Prep Kit was used for RNAseq, which was performed with a 500 ng of total RNA as input. Illumina HiSeq generated SE40 reads were aligned to the human reference genome version GRCh37.64 using TopHat v2.0.4 [49]. HTSeq-count v0.5.3p9 [50] to obtain read counts, which was normalised and tested for differential gene expression using the Bioconductor package *DESeq* v1.10.1 [51]. Multiple testing corrections were applied using the Benjamini–Hochberg method [52]. Genes were selected as differentially expressed such that FDR < 0.05. To address the impact of PBK on the AR transcriptome, the mRNA sequencing data from C4-2 cells was compared with our published androgen-regulated transcriptome data set (GSE18684) [31].

### Immunohistochemistry

Paraffin-embedded tissues were used to perform immunohistochemical staining to detect AR and Ki67. The methodology to detect AR [14] and Ki67 [15] were detected as described earlier, respectively. A pre-validated anti-PBK (Sigma HPA0055753, 1:250 dilution) was used.

### Human prostate tissue samples and hormone refractory (metastatic) tissue microarray construction

All samples were obtained from the Addenbrooke’s Cambridge University Hospital from patients who attended the Urology clinic. Hospital records were used to identify clinical data, which were contained in a prospectively maintained database (https://sbb.nds.ox.ac.uk/camprompt).

To obtain benign prostate tissue, holmium laser enucleation of the prostate was used. Primary PrCa specimen was obtained using Robotic assisted laparoscopic prostatectomy and castrate-resistant/hormone refractory cancer specimen was obtained using transurethral resection of the prostate (chTURP). For histology, tissue was fixed in 10% neutral buffered formalin. Haematoxylin and eosin (H&E) analysis was performed by the uropathologist on 5-micron thick sections. Patients with hormone refractory tumour/CRPC were defined as those who had a sustained rise in prostate specific antigen from nadir despite undergoing ADT treatment. Benign tissues obtained from prostatectomy or from primary PrCa (matched benign) was used as control. Formalin fixed paraffin-embedded tissue retrieved from the pathology archive was used as control for the chTURP specimens. The original H&E sections were reviewed by a uropathologist. Suitable areas of tumour tissue were included in the TMA were marked on the slides and corresponding paraffin blocks. Cores were removed from the donor blocks using a 2 mm skin biopsy punch and incorporated into recipient TMA blocks with pre-defined layout. For each sample two tumour cores and one benign adjacent core were incorporated. Immunohistochemistry was performed on 3.5-micron sections and was also blind reviewed blinded by the uropathologist to verify the pathology and suitability of the included tissue cores for scoring.

### Invasion assay

Both Boyden chamber and matrigel invasion assays were performed as previously described [14].

### Ex vivo prostate explant culture

PrCa tissue was obtained with informed consent in accordance with the institutional policy. Using a sterilised knife, the tissue was cut into 1–2 mm^3^ pieces, which were mounted on collagen cushions, kept on steel grids for 3 weeks to grow as explants. The tissue was treated as indicated with drugs in RPMI with 10% FBS, 1% penicillin, streptomycin and gentamycin. To prepare collagen cushions, 250 μl of collagen mix (rat tail collagen, RPMI medium, FBS and 10 × RPMI in the ratio of 7:1:1:1) was solidified on a nylon membrane. Tissue were harvested by fixation in formalin for 20 h and then transferred to 70% ethanol prior to paraffin embedding for immunohistochemistry.

### Scratch–wound assay

VCaP cells were selected for the scratch–wound assay on account of their strong adherence to tissue culture plates. Cells were transfected with 25 nM siRNA for 24 h using RNAiMAX reagent, by which time nearly confluent layers of VCaP cells had formed in a 24-well tissue culture plate. The scratch was introduced using a wound scratch instrument (Essen Bioscience) with a micropipette tip (1–2 µl capacity). The cells were washed with PBS, given fresh medium and treated with the indicated drugs. The culture plate was placed inside the Incucyte instrument and monitored for the required time.

### Statistical analysis

Statistical analyses were reported as mean ± SEM for continuous variables. We used Student’s *t* test where applicable. *P* value of 0.05 or less was considered significant. Appropriate nonparametric tests such as Mann–Whitney *U* test were used to analyse various datasets. Kaplan–Meier survival curves were generated by recursive partitioning analysis. KM curves are predictive of recurrence-free survival (corrected for the testing of multiple cutoffs, but not genes). *p < 0.05, **p < 0.01, ***p < 0.001.

## Accession numbers

The RNA-sequencing data have been submitted to Gene Expression Omnibus. It is available at the following links http://www.ncbi.nlm.nih.gov/geo/query/acc.cgi?token=ctgjggwqhlqbvod&acc=GSE63701 & http://www.ncbi.nlm.nih.gov/geo/query/acc.cgi?token=cparuuusjtcnlun&acc=GSE64341

## Electronic supplementary material

Fig S1 Warren

Fig S2 Warren

Fig S3 Warren

Fig S4 Warren

Fig S5 Warren

Table S1 Warren

Table S2 Warren

Table S3 Warren
