# Supplementary material for: A reciprocal feedback between the PDZ binding kinase and androgen receptor drives prostate cancer
Source: Oncogene. 2018 Sep 20;38(7):1136–50. doi: 10.1038/s41388-018-0501-z (PMC6514849; doi:10.1038/s41388-018-0501-z)
Supplement: Supplementary file 1 — Fig S1 Warren [file 41388_2018_501_MOESM1_ESM.pdf]

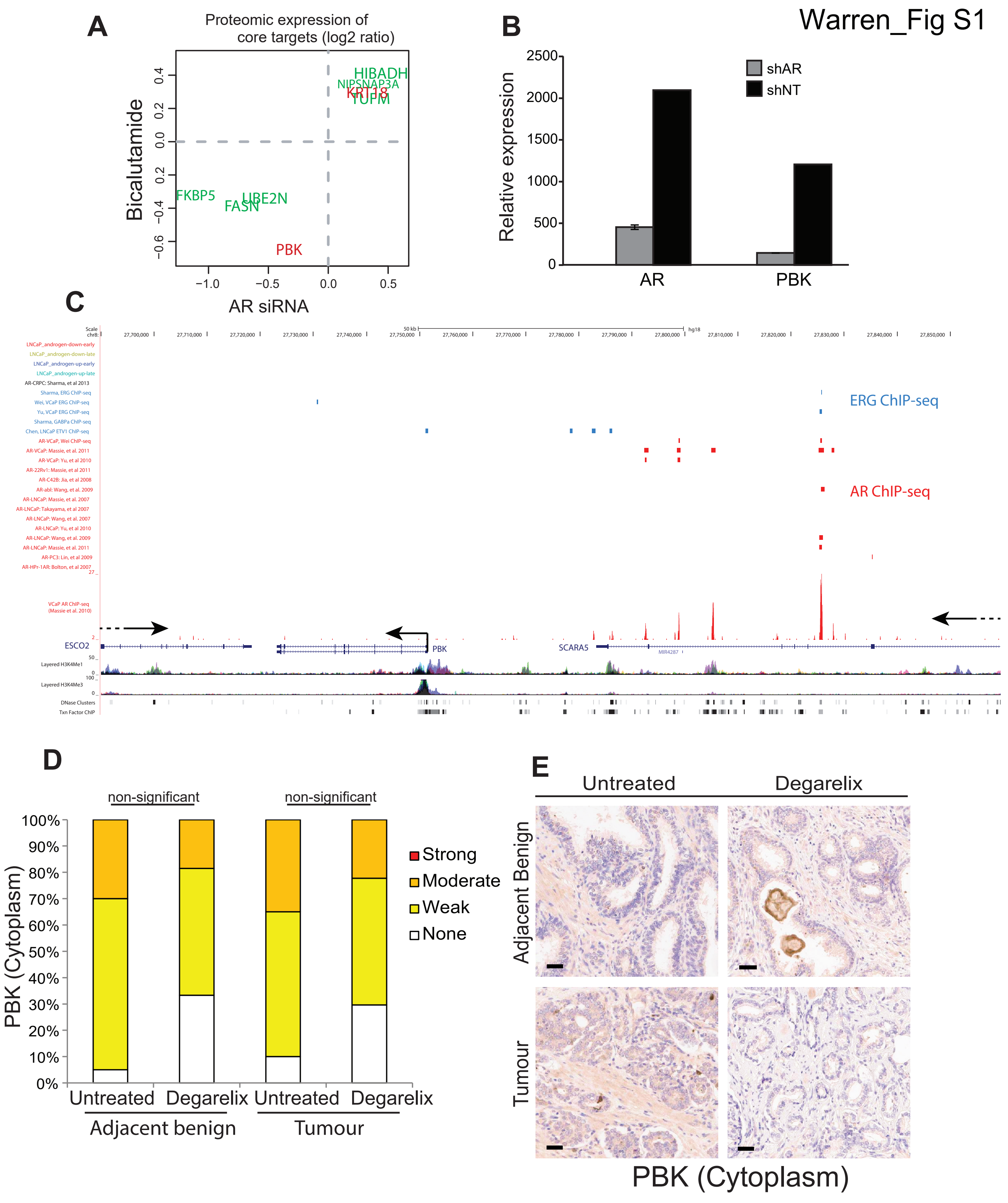

Supplementary figure 1. Androgen receptor regulates PBK expression in PrCa. **(A)** Scatterplots showing some of gene products regulated both by AR antagonist bicalutamide and AR knockdown. **(B)** Transcript expression of PBK in late-passage LNCaP cells (LP50) grown in hormone depleted media and transfected with AR shRNA or control shRNA, (GEO identifier GDS4113, gene expression omnibus). **(C)** Results of ChIP-seq experiments in multiple cell lines showing occurrence of AR and ERG binding sites within *PBK* gene in different PrCa cell lines. **(D-E)** Immuno histochemical staining score and representative photomicrographs of cytosolic PBK protein expression in tumours and untreated benign adjacent epithelia in patients with (n=27) or without (n=20) 7 days' treatment with the LHRH analogue degarelix. Shown above the bars are representative IHC images of corresponding samples from degarelix-treated and untreated patients. **Scale bars**=100 µm.
