# Supplementary material for: A reciprocal feedback between the PDZ binding kinase and androgen receptor drives prostate cancer
Source: Oncogene. 2018 Sep 20;38(7):1136–50. doi: 10.1038/s41388-018-0501-z (PMC6514849; doi:10.1038/s41388-018-0501-z)
Supplement: Supplementary file 2 — Fig S2 Warren [file 41388_2018_501_MOESM2_ESM.pdf]

Warren_Fig S2

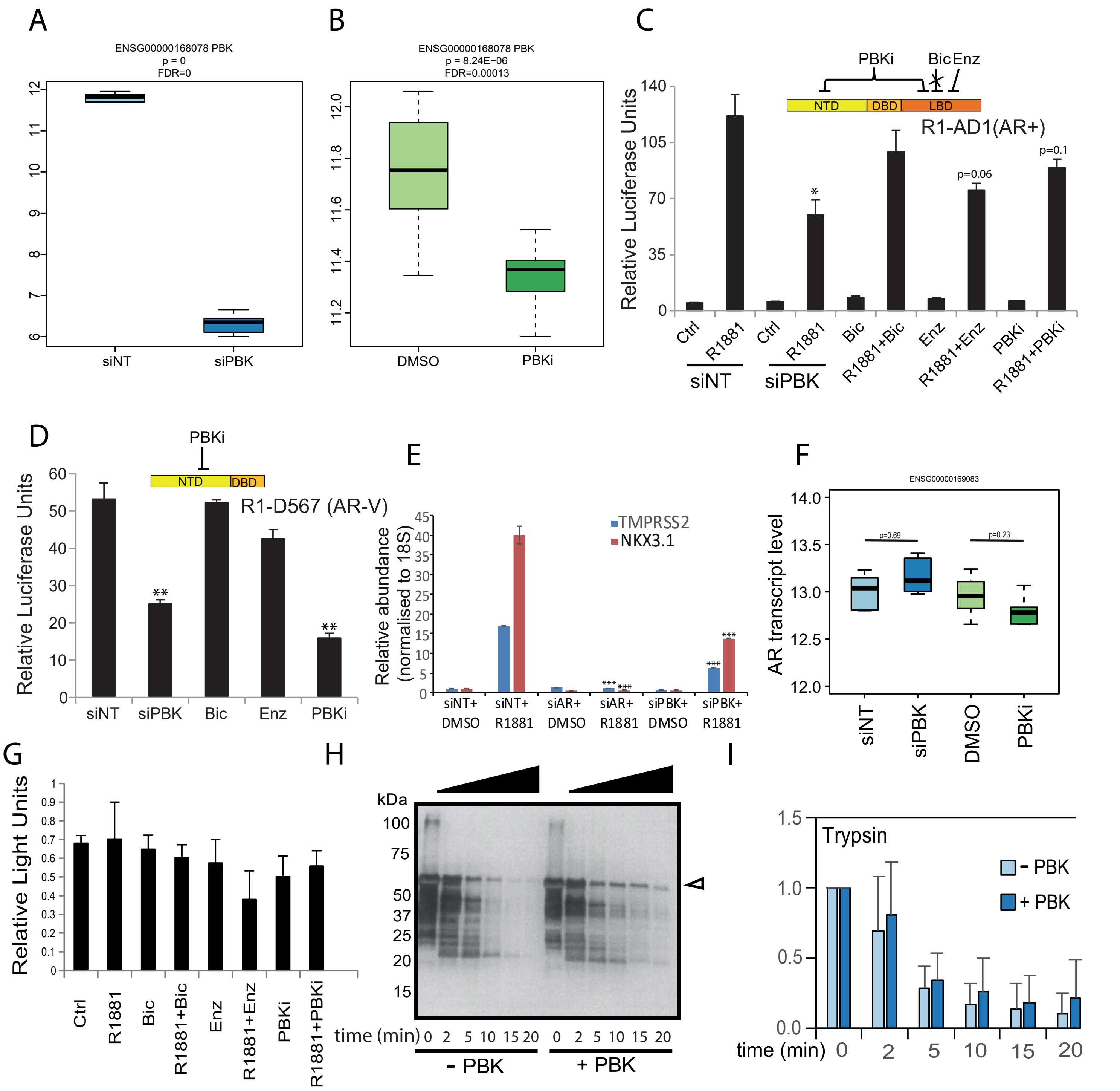

Supplementary figure 2. PBK regulates androgen receptor function. (**A**) Barplot shows result of RNAseq analysis of PBK transcript expression in C4-2 transiently transfected with siPBK for 72 hrs. (**B**) Barplot shows result of RNAseq analysis of PBK transcript expression in C4-2 treated with PBKi for 6 hrs. **(C)** Luciferase reporter assay showing AR transactivation potential in R1-AD1 & **(D)** R1-D567 cells. Cells were transfected with MMTV-Luc treated and siAR or siNT ± androgen (R1881); bars show mean ± SD (n=3). P values for two-sided Student's t-test. **(E)** qPCR of TMPRSS2 and NKX3.1 gene transcripts from C4-2 cells grown in charcoal treated media and transfected with siScr, siPBK or siAR (used a positive control) for 72 hr and treated ±R1881 for additional 24 hr; bars show mean ± SE (n=9). P values for two-sided Student's t-test. **(F)** Barplot shows result of the RNAseq analysis of *AR* gene transcript expression in C4-2 transiently transfected with siPBK or siNT for 72 hrs or treated with PBKi or DMSO control for 6 hrs. **(G)** Reporter assay on pLARS luciferase plasmid containing AR gene promoter. PC3 cells were transiently transfected with pLARS and treated with R1881 and/or Bic or PBKi for 24 hrs. Bars show mean with SD (n=3). Result shows normalised luciferase measurement. **(H)** Representative blot for ARNTD digested with trypsin in the absence or presence of recombinant PBK. Open triangle represents the full-length ARNTD. Proteolysis was performed for indicated times in the presence of trypsin (1ng). ARNTD fragments were detected on Western blots using the anti-androgen receptor antibody ab3510 corresponding to human AR amino acids 1-21 (N-terminal). **(I)** Quantitation of the ARNTD full-length polypeptide, after digestion with trypsin for two-independent experiments.
