# Supplementary material for: A reciprocal feedback between the PDZ binding kinase and androgen receptor drives prostate cancer
Source: Oncogene. 2018 Sep 20;38(7):1136–50. doi: 10.1038/s41388-018-0501-z (PMC6514849; doi:10.1038/s41388-018-0501-z)
Supplement: Supplementary file 3 — Fig S3 Warren [file 41388_2018_501_MOESM3_ESM.pdf]

Warren_Fig S3

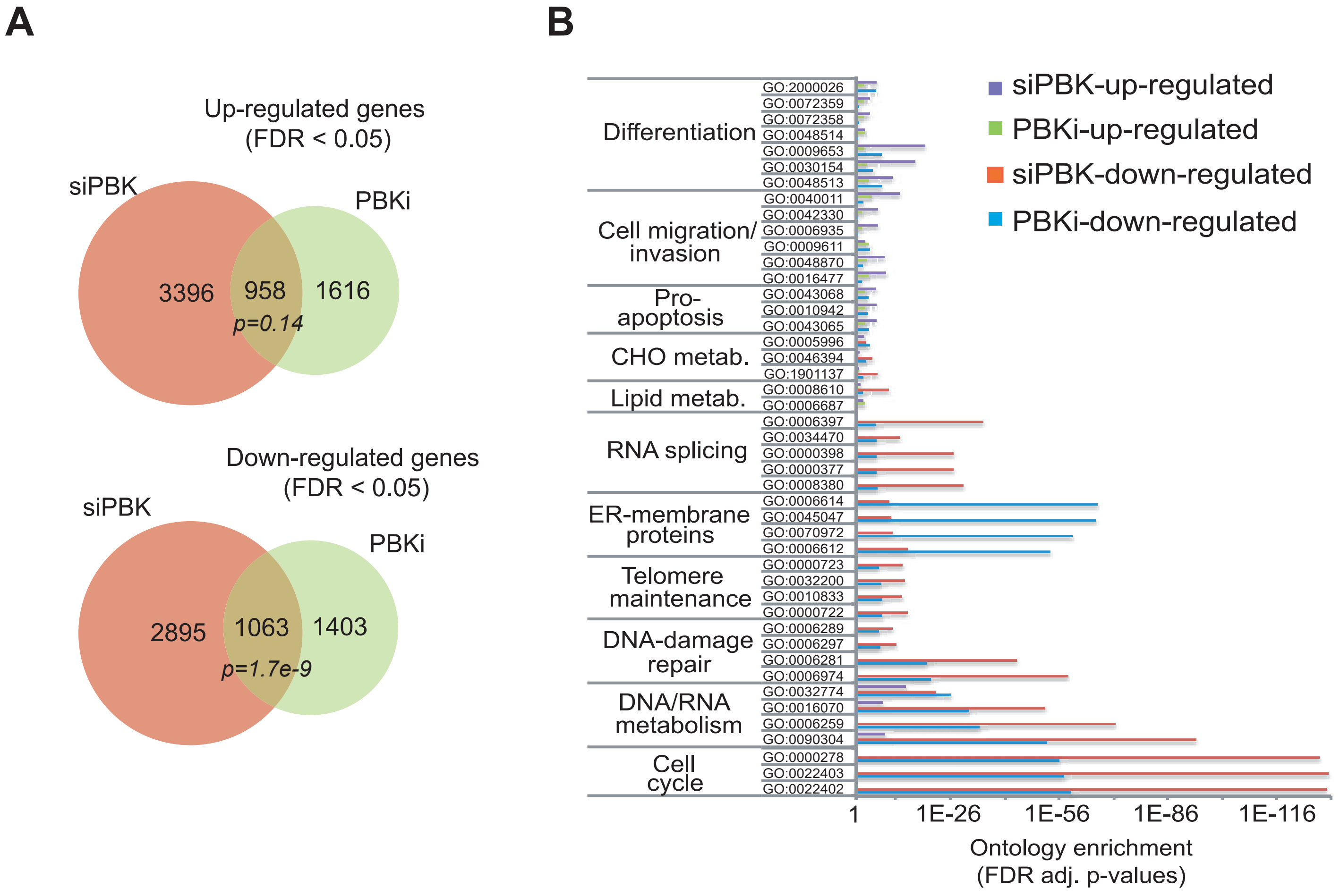


**Supplementary Figure 3.** The PBK regulated transcriptional program in PrCa. **(A)** Venn diagrams showing the overlap between differentially expressed genes between PBK knockdown (siPBK) and PBK inhibition (PBKi) conditions in C4-2 cells. Results from RNA-sequencing with six replicates per condition, differentially expressed genes were identified using an FDR adjusted p-value cut-off of 0.05. **(B)** Barplot summary of gene ontology term enrichment for genes differentially expressed following PBK inhibition or PBK knockdown (from panel A). Term enrichment are grouped by category and scores for differentially up- and down-regulated genes are shown separately to highlight specificity. FDR adjusted p-values from GOseq analysis are plotted from high (non-significant) to low (significant) on a log scale.
