# Supplementary material for: A reciprocal feedback between the PDZ binding kinase and androgen receptor drives prostate cancer
Source: Oncogene. 2018 Sep 20;38(7):1136–50. doi: 10.1038/s41388-018-0501-z (PMC6514849; doi:10.1038/s41388-018-0501-z)
Supplement: Supplementary file 4 — Fig S4 Warren [file 41388_2018_501_MOESM4_ESM.pdf]

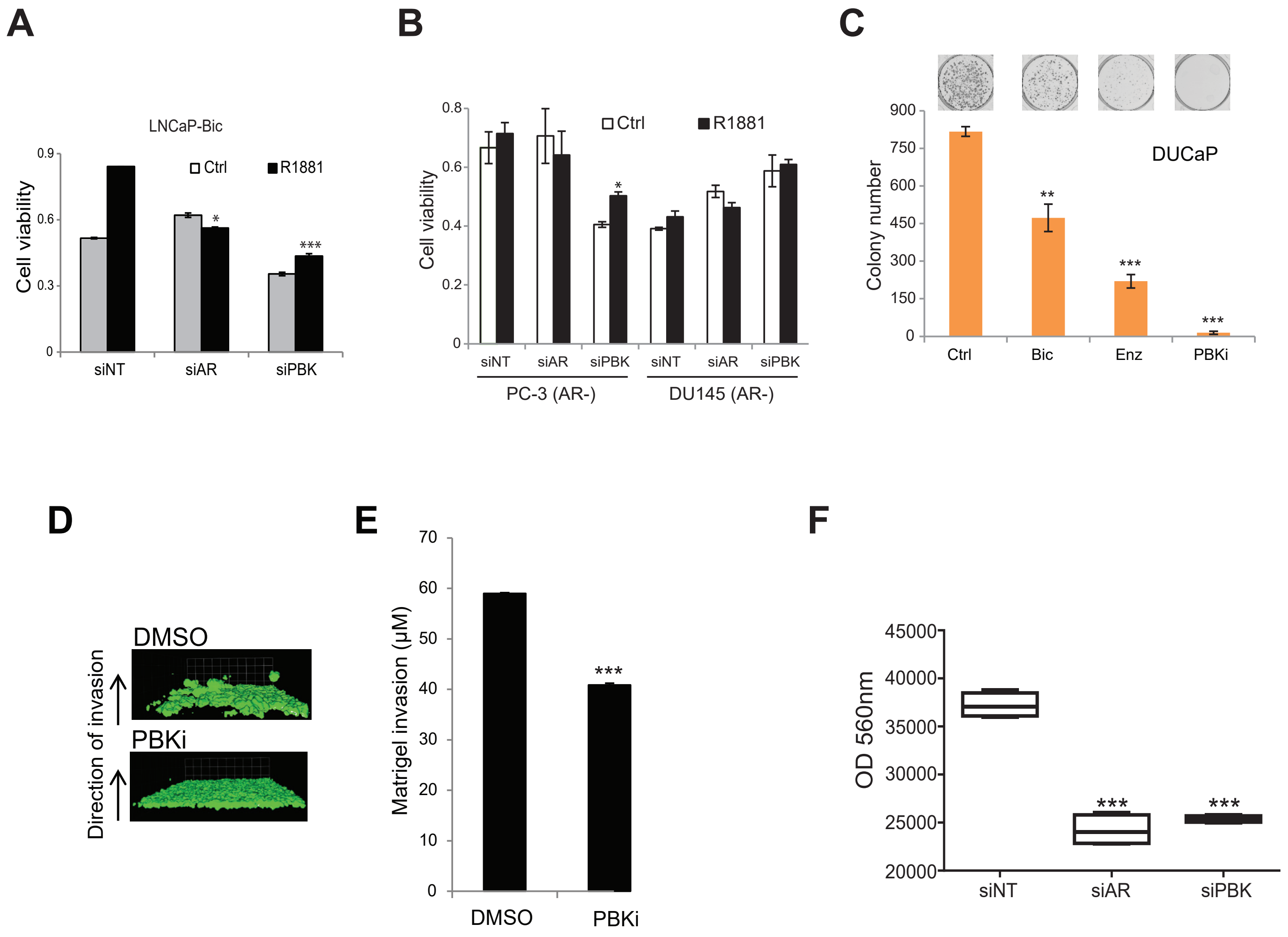


**Supplementary figure 4.** PBK inhibition represses PrCa cell growth. **(A-B)** MTS assay of LNCaP-Bic and **(B)** PC3 and DU145 cells transfected with indicated siRNA targeting AR (siAR) or PBK (siPBK) or non-targeting control (siNT). Cells were grown ± R1881 for 5 days or 95% confluence; bars show mean ± SD (n=3). **(C)** Clonogenic cell survival assay in DUCaP cell line treated for 14 days with Bicalutamide, Enzalutamide or PBKi (all at 10 μM); (n=3). P values by two-sided Student's t-test. (**D-E**) Matrigel based inverted invasion assay **D**) photomicrographs and **E**) quantification of GFP expressing C4-2 cells transiently transfected with indicated siRNA; bars show mean ± SEM (n=3), significance calculated by D'Agostino & Pearson omnibus normality test p<0.0001. **(F)** Barplot showing the result of trans-well migration assay showing the relative invasive ability of C4-2 cells upon AR and PBK knockdown with 25 nM siRNA; bars show ± SD (n=3).
