# Supplementary material for: A reciprocal feedback between the PDZ binding kinase and androgen receptor drives prostate cancer
Source: Oncogene. 2018 Sep 20;38(7):1136–50. doi: 10.1038/s41388-018-0501-z (PMC6514849; doi:10.1038/s41388-018-0501-z)
Supplement: Supplementary file 5 — Fig S5 Warren [file 41388_2018_501_MOESM5_ESM.pdf]

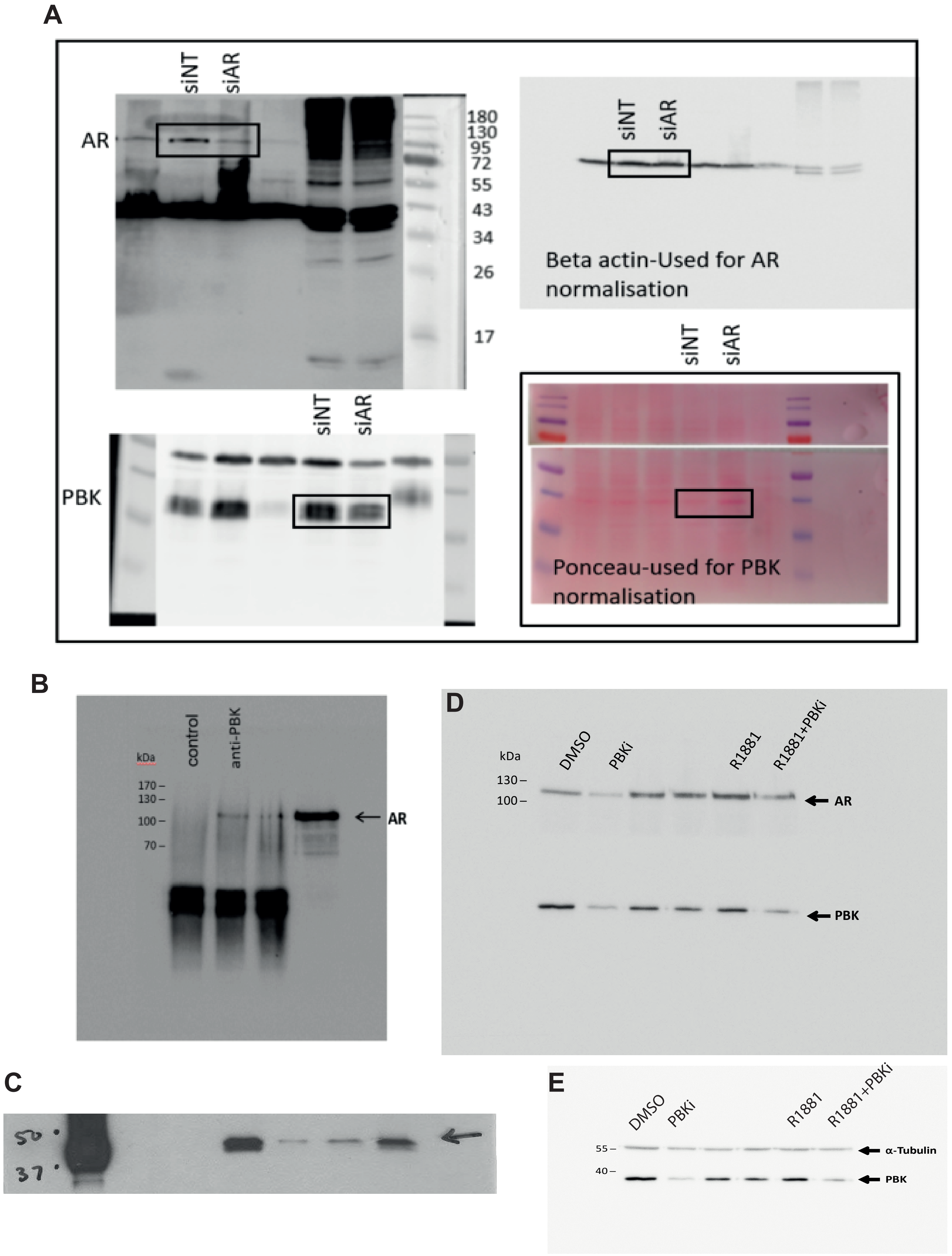


Uncropped images of the Western blot analyses shown in the main paper. (A) Figure 1B, (B) Figure 3C, (C) Figure 3E, (D-E) Figure 3G
