# Supplementary material for: A reciprocal feedback between the PDZ binding kinase and androgen receptor drives prostate cancer
Source: Oncogene. 2018 Sep 20;38(7):1136–50. doi: 10.1038/s41388-018-0501-z (PMC6514849; doi:10.1038/s41388-018-0501-z)
Supplement: Supplementary file 6 — Table S1 Warren [file 41388_2018_501_MOESM6_ESM.docx]

|  |  |  |  |  |
| --- | --- | --- | --- | --- |
| **Table S1: Changes in protein and transcript expression in C4-2 cells.** | | | | |
|  | **Changes at protein level** | | *Changes at transcript level* | |
| Gene Symbol | **AR knockdown** | **Bicalutamide** | *Bicalutamide* | *R1881* |
| GDF15 | **-1.51837** | **-0.6597226** | *-0.227923533* | *-0.415143733* |
| FKBP5 | **-1.086298** | **-0.3183259** | *-0.573575259* | *4.487376676* |
| RPIA | **-0.544733** | **-0.6896599** | *-0.129842826* | *0.026849106* |
| SAE1 | **-0.5346476** | **-0.6780719** | *-0.198972417* | *-0.289453845* |
| FASN | **-0.6920825** | **-0.3827015** | *-0.255154593* | *1.189846347* |
| PDCD4 | **-0.6948717** | **-0.3566055** | *0.151439297* | *-0.156217041* |
| NUDT5 | **-0.9369493** | **-0.1031469** | *-0.12146466* | *-0.073445637* |
| CD2AP | **-0.8960602** | **-0.1250064** | *-0.386107826* | *0.33759649* |
| TOMM34 | **-0.7441587** | **-0.2688168** | *-0.075991833* | *-0.044432032* |
| PAFAH1B2 | **-0.781979** | **-0.2058961** | *0.074716286* | *0.303232344* |
| MAP4 | **-0.6707469** | **-0.293359** | *-0.451778675* | *0.089519211* |
| PBK | **-0.3016705** | **-0.6461121** | *0.714999513* | *-0.271301347* |
| PRMT3 | **-0.7668988** | **-0.1746214** | *-0.260114543* | *-0.344996691* |
| ACACA | **-0.749358** | **-0.1909972** | *-0.11173193* | *0.552198164* |
| HLTF | **-0.8117183** | **-0.1140352** | *-0.175418566* | *-0.487290335* |
| GNL1 | **-0.4900306** | **-0.4208199** | *0.167492656* | *-0.190181759* |
| IMPDH2 | **-0.5742067** | **-0.3346073** | *0.202484497* | *-0.768892077* |
| RANBP1 | **-0.7548875** | **-0.142417** | *0.024731283* | *-0.2596232* |
| PDXK | **-0.7208527** | **-0.172994** | *-0.240681277* | *-0.165136371* |
| MAP1B | **-0.8085744** | **-0.08008794** | *0.434985823* | *-0.920428296* |
| GCLM | **-0.4687077** | **-0.4111955** | *-0.761741258* | *0.171850685* |
| TCEA1 | **-0.4598725** | **-0.4169624** | *-1.187994147* | *-0.450336352* |
| KLK3 | **-1.084699** | **0.2103888** | *-0.940738486* | *1.770164154* |
| DTD1 | **-0.3868828** | **-0.479955** | *-0.013786258* | *-0.1082058* |
| GMPPB | **-0.5898894** | **-0.2688168** | *-0.17335801* | *1.389937072* |
| UBE2N | **-0.4971712** | **-0.3382504** | *-0.37986782* | *0.429510239* |
| ACLY | **-0.7497302** | **-0.0740006** | *0.426099637* | *0.43912602* |
| NEK9 | **-0.7144215** | **-0.1078033** | *-0.176203572* | *0.025061925* |
| PEBP1 | **-0.661868** | **-0.1600404** | *0.168086767* | *0.151572204* |
| ABCE1 | **-0.400302** | **-0.4111955** | *0.010357263* | *-0.183699784* |
| VARS | **-0.4928418** | **-0.2986727** | *-0.429716806* | *-0.171805386* |
| CSDE1 | **-0.504839** | **-0.2827896** | *-0.307770517* | *-0.168226787* |
| CAD | **-0.5513323** | **-0.2310747** | *-0.332427903* | *-0.21726969* |
| C11orf54 | **-0.6564489** | **-0.1218632** | *-0.39047843* | *0.503618968* |
| STMN1 | **-0.389873** | **-0.3808218** | *0.061178891* | *0.174812208* |
| PEF1 | **-0.3531708** | **-0.4111955** | *0.190496266* | *0.140800612* |
| ME1 | **-0.4758185** | **-0.2792838** | *-0.166976673* | *0.164688001* |
| DNM1L | **-0.7603511** | **0.008630365** | *-0.32880293* | *1.17125856* |
| DARS | **-0.6694506** | **-0.07856363** | *0.33235679* | *-0.035095962* |
| THOP1 | **-0.5693722** | **-0.1697447** | *0.404253163* | *0.216327164* |
| TAGLN2 | **-0.6312327** | **-0.1078033** | *-0.361215667* | *0.275351148* |
| DPP3 | **-0.4330175** | **-0.3057884** | *0.168883076* | *0.109977213* |
| DHFR | **-0.5022367** | **-0.2327689** | *-0.105654478* | *-0.043987092* |
| SRI | **-0.5992188** | **-0.1328943** | *0.079930313* | *0.098138702* |
| DUSP23 | **-0.5304304** | **-0.1909972** | *0.171710597* | *-0.084439344* |
| CHMP2A | **-0.4763471** | **-0.2395661** | *-0.158182432* | *-0.113407255* |
| DUT | **-0.4513231** | **-0.2618807** | *0.062706866* | *-0.182631978* |
| RPA1 | **-0.7169837** | **0.007195495** | *0.138222863* | *-0.227053245* |
| CAND1 | **-0.604054** | **-0.1031469** | *0.135193762* | *-0.240003605* |
| NANS | **-0.5974245** | **-0.1046974** | *0.148054432* | *1.045362678* |
| PSMB4 | **-0.6845271** | **-0.01595754** | *-0.003429467* | *0.129134121* |
| TTLL12 | **-0.436585** | **-0.2567005** | *0.018420267* | *0.233120574* |
| EIF3J | **-0.6980182** | **0.007195495** | *-0.416414628* | *0.310370497* |
| PGAM1 | **-0.576356** | **-0.1031469** | *-0.178278794* | *0.085990636* |
| IPO4 | **-0.2551408** | **-0.4169624** | *-0.189594453* | *-0.010837325* |
| ARHGDIA | **-0.5342562** | **-0.1281563** | *0.202683823* | *0.22984219* |
| TARS | **-0.3429762** | **-0.3147326** | *-0.02881558* | *0.372796993* |
| TSTA3 | **-0.6239089** | **-0.03356954** | *0.214612946* | *0.341281931* |
| PRDX1 | **-0.4949964** | **-0.1616533** | *-0.024609523* | *-0.016409927* |
| CACYBP | **-0.6414192** | **-0.01304304** | *-0.494128177* | *-0.29528983* |
| PSMA4 | **-0.6595199** | **0.005759195** | *-0.015595467* | *-0.071771444* |
| NCAPG | **-0.2971287** | **-0.3529158** | *1.087528423* | *-0.056898982* |
| HCFC1 | **-0.09369543** | **-0.5542733** | *-0.230071862* | *-0.343098777* |
| GDI2 | **-0.6214131** | **-0.02620508** | *0.406732177* | *-0.165725295* |
| PTGES3 | **-0.7001579** | **0.05797008** | *-0.304748908* | *-0.037760598* |
| XPNPEP1 | **-0.4807351** | **-0.1600404** | *-0.108063183* | *0.299343789* |
| SMC4 | **-0.4321383** | **-0.2058961** | *-0.043931142* | *-0.108581288* |
| FXR1 | **-0.3694236** | **-0.2670797** | *-0.107505223* | *-0.32466382* |
| COMT | **-0.5273477** | **-0.1078033** | *-0.42852907* | *0.186568021* |
| PITPNB | **-0.5269139** | **-0.09387908** | *-0.051370872* | *-0.044471131* |
| EZR | **-0.5856757** | **-0.03356954** | *0.44273805* | *0.610232023* |
| TKT | **-0.4236208** | **-0.1909972** | *-0.300748077* | *-0.36672725* |
| WARS | **-0.39869** | **-0.2159149** | *-0.18462536* | *0.495775377* |
| HDGF | **-0.5397941** | **-0.0740006** | *0.09818021* | *0.00240193* |
| GAMT | **-0.5136845** | **-0.09850552** | *0.335419053* | *0.080481762* |
| APEH | **-0.429599** | **-0.1762507** | *0.071040463* | *0.081982898* |
| ESD | **-0.7905892** | **0.1890338** | *-0.048634717* | *-0.386518375* |
| EIF4B | **-0.3493961** | **-0.2498223** | *0.18973829* | *-0.6567029* |
| G6PD | **-0.3926978** | **-0.2042331** | *-0.006674903* | *0.322562236* |
| MTHFD1 | **-0.5411345** | **-0.0543923** | *0.40253249* | *0.092257696* |
| USP5 | **-0.3604701** | **-0.2327689** | *0.194274623* | *0.461043998* |
| GLO1 | **-0.5190371** | **-0.0740006** | *-0.12799688* | *0.223829871* |
| SRM | **-0.4367806** | **-0.153607** | *0.090883197* | *0.599589337* |
| EEF1A2 | **-0.4282045** | **-0.1616533** | *0.103161873* | *-0.445998092* |
| YWHAZ | **-0.5328053** | **-0.04245678** | *0.05699594* | *0.440228297* |
| EEF1A1 | **-0.2331314** | **-0.3364277** | *-0.541311557* | *-0.681885842* |
| UBA1 | **-0.4457725** | **-0.1202942** | *0.339180887* | *0.005722176* |
| FKBP4 | **-0.4308226** | **-0.1328943** | *0.109095283* | *-0.000114253* |
| HSP90AB1 | **-0.3887108** | **-0.1746214** | *0.028290484* | *-0.45749321* |
| EIF5B | **-0.4137374** | **-0.1488006** | *0.165768127* | *0.682495939* |
| HSP90AA1 | **-0.4685701** | **-0.08926734** | *-0.141723324* | *-0.55416444* |
| PPA1 | **-0.4694921** | **-0.0877334** | *-0.580378213* | *-0.226542225* |
| ACTR1A | **-0.3507917** | **-0.2058961** | *0.125965787* | *0.468874806* |
| SERBP1 | **-0.2079633** | **-0.3473988** | *-0.175080157* | *-0.257327671* |
| EIF5A | **-0.4518754** | **-0.1015982** | *0.037858337* | *0.123987048* |
| HMGB3 | **-0.2894652** | **-0.2636116** | *0.262836634* | *0.110710029* |
| PFDN2 | **-0.5189341** | **-0.03209365** | *-0.050396235* | *0.220070208* |
| EIF2A | **-0.3970992** | **-0.153607** | *-0.223452037* | *-0.2984885* |
| PSMB6 | **-0.4913472** | **-0.05589118** | *-0.209440313* | *0.29771196* |
| SARS | **-0.3804247** | **-0.1665027** | *-0.340176323* | *-0.329863243* |
| EIF5 | **-0.3928981** | **-0.153607** | *-0.170706323* | *-0.432497796* |
| ALDOA | **-0.520454** | **-0.02326974** | *0.048120861* | *0.007999261* |
| TAF9 | **-0.4913313** | **-0.05139919** | *-0.589016865* | *-0.100686755* |
| PSMB2 | **-0.3655371** | **-0.1713684** | *-0.39396994* | *0.313915324* |
| EIF3M | **-0.3716779** | **-0.1648844** | *-0.03367846* | *-0.13807059* |
| CLIC1 | **-0.3986218** | **-0.1328943** | *0.241317323* | *0.470184572* |
| CCDC124 | **-0.4273536** | **-0.1031469** | *-0.094227209* | *0.380550535* |
| PSMA5 | **-0.4027086** | **-0.1265805** | *-0.304924217* | *0.323786357* |
| FDPS | **-0.4635342** | **-0.06491752** | *-0.014021573* | *1.167857645* |
| CMPK1 | **-0.4521413** | **-0.06793883** | *0.131418783* | *0.016289028* |
| ENO1 | **-0.4468576** | **-0.07248279** | *0.291397553* | *-0.003302342* |
| UCHL5 | **-0.4137404** | **-0.1046974** | *-0.322424652* | *0.572365075* |
| MAPK1 | **-0.2771001** | **-0.2412704** | *0.065322642* | *0.045647685* |
| MAT2B | **-0.4309807** | **-0.08620108** | *-0.179107287* | *0.358919863* |
| PSMC2 | **-0.3964743** | **-0.1187269** | *0.007325453* | *0.323822415* |
| VCL | **-0.4773854** | **-0.03356954** | *-0.098477157* | *0.804942511* |
| PSMA1 | **-0.4134835** | **-0.09696173** | *0.170156232* | *-0.263003814* |
| RPL18A | **-0.290908** | **-0.2192699** | *-0.417389257* | *-0.256344812* |
| HMGB1 | **-0.4274505** | **-0.08161378** | *0.173009169* | *-0.426499222* |
| SART3 | **-0.4432709** | **-0.06491752** | *0.199280141* | *-0.080431069* |
| ABCF3 | **-0.4036775** | **-0.1031469** | *0.250640875* | *0.381576959* |
| TBCA | **-0.3279645** | **-0.1762507** | *-0.19152736* | *0.097079875* |
| DNM2 | **-0.4790029** | **-0.02180435** | *0.523326766* | *0.625435569* |
| EIF1AY | **-0.4720668** | **-0.02767495** | *-0.157267603* | *0.067805624* |
| PSMC3 | **-0.4649951** | **-0.03209365** | *0.283994507* | *0.237222376* |
| STAT1 | **-0.1786657** | **-0.3165281** | *-0.256103774* | *-0.196254011* |
| EPRS | **-0.3913465** | **-0.1015982** | *-0.05603696* | *-0.472928166* |
| AARS | **-0.322093** | **-0.1665027** | *0.061832443* | *-0.205514614* |
| TUBB | **-0.4340391** | **-0.0543923** | *0.35379037* | *0.03077597* |
| APRT | **-0.4942796** | **0.01720924** | *0.111287397* | *-0.190592073* |
| PFAS | **-0.3829368** | **-0.09387908** | *-0.425583391* | *-0.130697875* |
| PRDX2 | **-0.4592417** | **-0.01595754** | *0.061172828* | *-0.155889928* |
| AKR1A1 | **-0.4146553** | **-0.05889372** | *0.11184311* | *0.19751206* |
| PSMA7 | **-0.4478441** | **-0.02180435** | *0.028896617* | *0.346555138* |
| AHCY | **-0.3998939** | **-0.06491752** | *-0.145279397* | *-0.091387038* |
| PDCD10 | **-0.436568** | **-0.02767495** | *-0.131602976* | *0.64397669* |
| PAFAH1B3 | **-0.4149692** | **-0.04841219** | *0.05365946* | *0.135501986* |
| YARS | **-0.2308592** | **-0.2310747** | *-0.14457243* | *-0.20667096* |
| PSAT1 | **-0.3020077** | **-0.1584293** | *-0.5662392* | *-0.835891568* |
| NUDC | **-0.4454318** | **-0.011588** | *0.00373852* | *0.002686664* |
| GAPDH | **-0.365826** | **-0.08926734** | *0.889561037* | *-0.074334956* |
| RARS | **-0.3027717** | **-0.1520031** | *-0.111278473* | *0.067370833* |
| STIP1 | **-0.3531344** | **-0.100051** | *0.059476827* | *0.028829112* |
| CCT3 | **-0.2887339** | **-0.1600404** | *0.02231069* | *-0.208040026* |
| PPP2R1A | **-0.2882628** | **-0.1568201** | *0.254995517* | *0.088952191* |
| UPF1 | **-0.2661411** | **-0.1778817** | *0.195553916* | *0.250652292* |
| PSMB5 | **-0.4192055** | **-0.02473671** | *0.055622653* | *0.063058684* |
| PSMD14 | **-0.3984426** | **-0.04245678** | *-0.011347333* | *0.255566094* |
| G3BP1 | **-0.2484293** | **-0.1860649** | *-0.128416819* | *-0.174227547* |
| RPS10 | **-0.4222449** | **-0.011588** | *0.005911603* | *0.039258733* |
| YWHAE | **-0.358866** | **-0.07248279** | *-0.59520484* | *0.002251706* |
| EEF2 | **-0.3948448** | **-0.03061928** | *-0.152512767* | *-0.150495993* |
| PSMB3 | **-0.4392752** | **0.01435528** | *0.09013941* | *0.212288991* |
| COMMD3 | **-0.380071** | **-0.04097178** | *0.10332334* | *-0.199277709* |
| CKB | **-0.4374291** | **0.01720924** | *0.634692007* | *0.311033532* |
| NACA | **-0.3939555** | **-0.02473671** | *0.014528537* | *-0.263344739* |
| NUDCD2 | **-0.4326089** | **0.01435528** | *-0.336394052* | *-0.08179881* |
| DYNC1H1 | **-0.4155056** | **0** | *0.60710682* | *-0.324112892* |
| SMC2 | **-0.180447** | **-0.2310747** | *-0.01639282* | *0.203846223* |
| CNDP2 | **-0.5329634** | **0.1269729** | *-0.143534193* | *0.578149948* |
| PCMT1 | **-0.3980066** | **-0.007231562** | *-0.37023712* | *0.119942719* |
| PARK7 | **-0.3380874** | **-0.06190243** | *0.149076387* | *0.305671817* |
| HSPA4L | **-0.3321257** | **-0.06642739** | *0.252673716* | *-0.669133399* |
| COPB1 | **-0.3752199** | **-0.01741709** | *0.395759743* | *0.290276116* |
| PDCD6IP | **-0.3274828** | **-0.06340914** | *-0.04035286* | *-0.287877791* |
| FARSA | **-0.3169792** | **-0.07096649** | *0.354501382* | *0.364128235* |
| ACTR2 | **-0.3019147** | **-0.08314123** | *0.096202193* | *0.105923498* |
| PCNA | **-0.353649** | **-0.03061928** | *-0.091024459* | *-0.502122095* |
| AKR7A2 | **-0.3835962** | **0.002882472** | *-0.18426522* | *-0.130429373* |
| PAICS | **-0.3947388** | **0.01435528** | *0.042736493* | *-0.173049629* |
| QARS | **-0.3968099** | **0.01720924** | *0.228156523* | *-0.077503333* |
| SNX2 | **-0.2691071** | **-0.1078033** | *-0.56975828* | *-0.069398951* |
| PYCRL | **-0.4587193** | **0.08542464** | *-0.024526451* | *0.484415283* |
| TPD52L2 | **-0.4169714** | **0.04404436** | *0.029956565* | *0.018913333* |
| UROD | **-0.3578331** | **-0.01304304** | *0.431300563* | *-0.299532173* |
| CAP1 | **-0.2705255** | **-0.100051** | *-0.099649203* | *0.228416028* |
| RPS3 | **-0.1897406** | **-0.1795146** | *-0.211927917* | *-0.249439791* |
| SEC31A | **-0.2242232** | **-0.142417** | *0.071729028* | *0.127603291* |
| PSMA3 | **-0.3278327** | **-0.03800636** | *-0.29433322* | *0.19883805* |
| PSMA6 | **-0.2817571** | **-0.08314123** | *0.020434517* | *0.763428549* |
| RPS16 | **-0.2448623** | **-0.1155975** | *-0.121003497* | *0.116391759* |
| UBE2M | **-0.2890282** | **-0.06945187** | *0.034179247* | *0.292629646* |
| PAF1 | **-0.1842156** | **-0.1681228** | *0.162013363* | *0.329385792* |
| USP10 | **-0.236496** | **-0.1140352** | *-0.237714483* | *0.719619322* |
| NASP | **-0.3102781** | **-0.0394883** | *0.095206523* | *-0.108863228* |
| ISOC1 | **-0.3325028** | **-0.01595754** | *-0.502141503* | *0.005969698* |
| OSTF1 | **-0.3511082** | **0.008630365** | *0.02571657* | *0.537561395* |
| CSE1L | **-0.3189287** | **-0.02180435** | *-0.124869503* | *-0.156191363* |
| PPIA | **-0.3194867** | **-0.02034045** | *0.208116037* | *-0.183019504* |
| EXOSC5 | **-0.3681806** | **0.02856912** | *-0.088911414* | *0.015159233* |
| ACP1 | **-0.3089619** | **-0.03061928** | *-0.245841747* | *-0.248283217* |
| GLOD4 | **-0.2762327** | **-0.06039729** | *0.029657847* | *-0.017847958* |
| TUBA4A | **-0.2949129** | **-0.04097178** | *0.444180284* | *-0.239654958* |
| PSMA2 | **-0.2988049** | **-0.03652584** | *0.056369033* | *0.264563888* |
| ACTN1 | **-0.3258557** | **-0.007231562** | *-0.02563773* | *0.11481909* |
| USP14 | **-0.2555712** | **-0.07704102** | *-0.0811789* | *-0.264253145* |
| GINS3 | **-0.2983357** | **-0.03356954** | *-0.146089575* | *0.284454177* |
| EIF2S3 | **-0.2035224** | **-0.1250064** | *-0.169243063* | *-0.572997499* |
| PRMT1 | **-0.2155312** | **-0.1109159** | *-0.005439546* | *-0.182998687* |
| COPA | **-0.3855536** | **0.06350289** | *0.008188803* | *0.014766038* |
| AP3D1 | **-0.2792797** | **-0.0394883** | *-0.123043175* | *0.137319536* |
| MTAP | **-0.3197176** | **0.002882472** | *-0.315158269* | *0.04032102* |
| TBCB | **-0.4333386** | **0.1176951** | *0.345149223* | *-0.034657722* |
| SET | **-0.3295414** | **0.01863419** | *-0.104482367* | *-0.287823016* |
| RPL21 | **-0.1246797** | **-0.1811494** | *-0.065330697* | *-0.148632895* |
| RPL7 | **-0.2205999** | **-0.08161378** | *-0.073007204* | *-0.628616483* |
| PA2G4 | **-0.2413472** | **-0.06039729** | *0.258850443* | *0.183155582* |
| RNH1 | **-0.1984801** | **-0.1031469** | *0.354467533* | *0.375365856* |
| SERPINB6 | **-0.2693535** | **-0.02914632** | *0.117752437* | *0.168175576* |
| MTHFD1L | **0.2378937** | **-0.5353317** | *-0.026216232* | *-0.130483234* |
| DRG2 | **-0.1876057** | **-0.1093588** | *0.172891416* | *0.33097947* |
| PABPC1 | **-0.2572865** | **-0.03061928** | *-0.057421273* | *-0.465413826* |
| RUVBL2 | **-0.3015758** | **0.02573757** | *0.107086036* | *0.147563898* |
| C12orf10 | **-0.2220217** | **-0.0439433** | *0.10578876* | *-0.013003327* |
| LARS | **-0.2272587** | **-0.03800636** | *0.052433073* | *-0.428152909* |
| RPS7 | **-0.2839848** | **0.02715413** | *-0.2330385* | *-0.210091695* |
| MAPK14 | **-0.0851341** | **-0.1713684** | *-0.251374024* | *0.122603196* |
| RPSA | **-0.1537232** | **-0.09541958** | *-0.561446344* | *-0.253509066* |
| API5 | **-0.1171126** | **-0.1202942** | *-0.26473023* | *0.025170588* |
| EIF3K | **-0.377262** | **0.1401242** | *-0.238071087* | *-0.232284143* |
| UNC45A | **-0.2088387** | **-0.02180435** | *0.372886527* | *-0.014691312* |
| RPS20 | **-0.1770109** | **-0.04990488** | *-0.00214224* | *0.054967029* |
| PCBP2 | **-0.1578816** | **-0.06793883** | *0.309359307* | *-0.525524972* |
| STUB1 | **-0.1665259** | **-0.05739162** | *0.1954137* | *-0.058851419* |
| HSPA8 | **-0.2321183** | **0.008630365** | *-0.671971833* | *-0.216950293* |
| EIF3B | **-0.1330374** | **-0.0846703** | *-0.04624788* | *-0.04905648* |
| YWHAB | **-0.3544956** | **0.1388144** | *-0.287665193* | *-0.104563789* |
| APOA1BP | **-0.2722552** | **0.06212175** | *-0.1206151* | *-0.11011952* |
| ASNA1 | **-0.03685205** | **-0.1713684** | *-0.154399554* | *0.214517743* |
| HIST1H4E | **0.3190369** | **-0.5269924** | *0.021667928* | *0.042344433* |
| RPS29 | **-0.2356528** | **0.02856912** | *0.13608698* | *-0.808320745* |
| MYL6 | **-0.2170538** | **0.01006364** | *0.31869943* | *0.254601829* |
| SSB | **-0.3876559** | **0.1865006** | *0.056626593* | *-0.028103205* |
| RFC4 | **-0.06889464** | **-0.1265805** | *-0.054446133* | *-0.381511067* |
| GNB2L1 | **-0.1767462** | **-0.004334621** | *0.148064827* | *0.006043821* |
| ARPC4 | **-0.1884757** | **0.01149566** | *-0.180747179* | *0.458857314* |
| RPS24 | **-0.3041835** | **0.1388144** | *-0.139452343* | *0.046549199* |
| RPL29 | **-0.1635129** | **0.005759195** | *-0.04100384* | *-0.168490734* |
| RPS25 | **-0.2026626** | **0.04544289** | *0.025880087* | *-0.167920007* |
| NT5C | **-0.3382406** | **0.1814206** | *0.21730322* | *0.237229777* |
| RPS6 | **-0.2081277** | **0.06626152** | *-0.07201862* | *0.056596677* |
| RPS19 | **-0.1785485** | **0.04963072** | *0.134596483* | *0.039336048* |
| BUB3 | **0.1593485** | **-0.2845459** | *-0.016686023* | *-0.232487309* |
| NOLC1 | **-0.2944657** | **0.169925** | *-0.626397891* | *0.146586223* |
| RPLP0 | **-0.2206345** | **0.09761087** | *-0.122033005* | *-0.403324526* |
| PABPC4 | **-0.159565** | **0.04124304** | *0.033424557* | *-0.785825563* |
| TXNL1 | **-0.3946837** | **0.2785796** | *-0.081287736* | *-0.204670646* |
| TYMP | **0.2307365** | **-0.332789** | *0.999654217* | *0.011226449* |
| VCP | **-0.1997654** | **0.1003049** | *-0.205383357* | *0.433387672* |
| RPL23 | **-0.1055412** | **0.008630365** | *0.234154617* | *0.079303154* |
| DEGS1 | **-0.1020234** | **0.005759195** | *-0.068929457* | *0.864041199* |
| CAPRIN1 | **-0.3906042** | **0.3033423** | *0.312081815* | *-0.404831507* |
| RPS9 | **-0.1356025** | **0.05241581** | *-0.19787154* | *-0.099389876* |
| RPL22L1 | **-0.1433567** | **0.06073913** | *-0.616432021* | *0.262895149* |
| PRDX5 | **-0.1110876** | **0.03984026** | *0.102457673* | *-0.164714382* |
| PPIL1 | **0.1496295** | **-0.2192699** | *-0.121281819* | *-0.224888275* |
| SAR1A | **-0.3223254** | **0.254594** | *0.30257648* | *0.098812552* |
| GRHPR | **-0.1754449** | **0.1083571** | *0.022590607* | *0.054273026* |
| RPS5 | **-0.04757865** | **-0.01013442** | *-0.159709927* | *-0.047902116* |
| POLR1D | **-0.08035775** | **0.02998291** | *0.48281484* | *-0.925136323* |
| NUP35 | **0.3235983** | **-0.3695945** | *-0.512634935* | *0.14948829* |
| SND1 | **-0.08166586** | **0.03703076** | *0.37931107* | *0.519712593* |
| RPL31 | **-0.09194484** | **0.04823624** | *0.155937443* | *0.048726373* |
| DCXR | **-0.1461775** | **0.105678** | *0.07081266* | *0.778874411* |
| TPD52 | **-0.3266295** | **0.2939589** | *0.019824058* | *1.246767249* |
| RAC1 | **0.04516613** | **-0.07248279** | *-0.074163907* | *-0.651878875* |
| KHSRP | **-0.0159661** | **-0.008682217** | *0.126036987* | *-0.091490314* |
| ACTB | **0.09769783** | **-0.1187269** | *0.297686527* | *0.105384809* |
| RPL24 | **-0.09295785** | **0.1070183** | *-0.095056357* | *-0.079155913* |
| JUP | **0.231416** | **-0.1762507** | *0.129838063* | *-0.361776808* |
| PYCR2 | **0.2251159** | **-0.1632679** | *0.179604527* | *-0.062866432* |
| HNRNPC | **0.2890544** | **-0.2260036** | *0.275349607* | *-0.194193149* |
| KIF5B | **0.1373611** | **-0.06340914** | *-0.685289711* | *-0.182379455* |
| RAE1 | **0.2143953** | **-0.1376478** | *-0.292016765* | *-0.178382044* |
| ENDOG | **0.3912042** | **-0.3129393** | *0.178820578* | *0.372933553* |
| POLR2A | **0.464839** | **-0.3827015** | *0.06822365* | *-0.370212447* |
| DDX3X | **0.1589002** | **-0.06190243** | *-0.038480589* | *0.107510796* |
| LSS | **0.1674459** | **-0.06793883** | *0.123379916* | *1.691964224* |
| RPL10 | **-0.2413595** | **0.3448285** | *-0.049851374* | *0.119810176* |
| RPL32 | **-0.1231555** | **0.230203** | *-0.5292895* | *0.244638934* |
| CPSF3 | **0.2260714** | **-0.1155975** | *0.22319152* | *-0.283213467* |
| PC | **0.3052942** | **-0.1926451** | *-0.057622087* | *0.06197084* |
| NONO | **0.2005826** | **-0.08620108** | *-0.022852345* | *-0.435432245* |
| NPM1 | **0.2073027** | **-0.08620108** | *0.19011178* | *-0.554909791* |
| DBN1 | **0.532901** | **-0.4054514** | *0.194203527* | *-0.287814367* |
| GFM2 | **0.2015985** | **-0.06793883** | *-0.19046165* | *-0.256697877* |
| OPA1 | **0.06071323** | **0.0745054** | *-0.197651145* | *-0.070714787* |
| RPL3 | **0.06640337** | **0.07176267** | *-0.00241824* | *0.023901904* |
| DDX17 | **0.2091904** | **-0.05889372** | *0.2082723* | *-0.154573613* |
| SDHB | **0.1158899** | **0.03843611** | *0.187742863* | *-0.181953828* |
| LYPLA1 | **0.1306886** | **0.02715413** | *-1.121660378* | *-0.181119561* |
| GFM1 | **0.1000159** | **0.05797008** | *-0.02591164* | *0.618865648* |
| MFN1 | **0.5522584** | **-0.390245** | *0.11519861* | *0.539187837* |
| SF3B1 | **0.1317894** | **0.04544289** | *0.104779773* | *-0.191765975* |
| ELAVL1 | **0.1824985** | **0.002882472** | *-0.10224671* | *0.262741898* |
| NUP160 | **0.153601** | **0.03843611** | *-0.07380079* | *-0.194301276* |
| SKIV2L2 | **0.2297158** | **-0.02914632** | *0.424742427* | *-0.551846337* |
| GAA | **0.1668687** | **0.04404436** | *0.120319517* | *-0.038532176* |
| CALR | **0.1516961** | **0.06212175** | *0.780918427* | *-0.134777247* |
| HNRNPU | **0.1972233** | **0.01720924** | *0.020548921* | *-0.315132885* |
| SON | **0.3490811** | **-0.134477** | *-0.041508863* | *-0.154311154* |
| COX4I1 | **0.2631887** | **-0.04841219** | *0.129208367* | *0.099896307* |
| FBL | **0.1428647** | **0.07724293** | *0.03960719* | *-0.50132799* |
| NUP155 | **0.1746853** | **0.04684024** | *-0.060070443* | *-0.146156338* |
| PCK2 | **0.4151106** | **-0.1877071** | *-0.001895253* | *-0.544445628* |
| MRPL4 | **0.3806712** | **-0.1520031** | *-0.164507695* | *0.051837889* |
| MYBBP1A | **0.211094** | **0.02715413** | *0.118699638* | *-0.290320081* |
| SFXN3 | **0.09967855** | **0.1479579** | *0.019878629* | *-0.269318328* |
| ADAR | **0.2456366** | **0.005759195** | *-0.01792032* | *-0.419993549* |
| PELP1 | **0.2282979** | **0.02715413** | *0.090283617* | *-0.108062533* |
| HIC2 | **0.05773858** | **0.1991226** | *0.003721179* | *-0.094473918* |
| SUMO1 | **0.3491166** | **-0.09080292** | *-0.001823779* | *-0.209667357* |
| TMEM126A | **0.4746248** | **-0.2125676** | *-0.506486533* | *-0.092037702* |
| SPCS3 | **0.1536984** | **0.1110314** | *-0.22109825* | *0.844196545* |
| FOLH1 | **0.4017513** | **-0.1313132** | *0.51127666* | *-1.099061165* |
| MYEF2 | **0.1893885** | **0.08270261** | *0.081571468* | *-0.027360966* |
| RAB7A | **0.2392956** | **0.03280615** | *0.18576835* | *-0.391208235* |
| TIMM23 | **0.2760069** | **0.007195495** | *-0.347207205* | *0.601076276* |
| TARS2 | **0.2702278** | **0.02005774** | *-0.003319026* | *-0.155563437* |
| SEH1L | **0.2718314** | **0.02147971** | *-0.059932012* | *-0.246031042* |
| RPL19 | **0.2811835** | **0.01292609** | *0.020598923* | *-0.014705325* |
| AP2A1 | **0.2817005** | **0.02147971** | *0.459820194* | *0.178786427* |
| DHODH | **0.2147387** | **0.08949816** | *-0.077280392* | *0.238800413* |
| MRPL10 | **0.2961336** | **0.008630365** | *0.129849611* | *0.03099055* |
| AK3 | **0.2244629** | **0.08406419** | *0.35399124* | *-0.256436906* |
| PPT1 | **0.2970529** | **0.01292609** | *0.10645393* | *-0.458378578* |
| U2AF2 | **0.244382** | **0.07038926** | *-0.061573403* | *-0.232449491* |
| PDIA6 | **0.1189136** | **0.1966071** | *-0.042234613* | *0.351684584* |
| TMED4 | **0.1299071** | **0.1915626** | *-0.1318564* | *-0.097960956* |
| PITRM1 | **0.2939026** | **0.02856912** | *0.145749575* | *0.418080345* |
| ERP29 | **0.3012676** | **0.02147971** | *-0.969418901* | *-0.38811422* |
| DDX23 | **0.1500914** | **0.1750454** | *0.258103472* | *-0.12365157* |
| PRKCSH | **0.2686611** | **0.05797008** | *-0.239327457* | *-0.221341366* |
| ARL2 | **0.2287086** | **0.09895846** | *-0.072791987* | *-0.217594587* |
| CLPB | **0.2661386** | **0.06763869** | *0.022258535* | *0.009652472* |
| CSTF3 | **0.2322484** | **0.102994** | *-0.1123878* | *-0.433631163* |
| TMEM109 | **0.273337** | **0.06488286** | *-0.062932407* | *0.212075257* |
| DKC1 | **0.2588263** | **0.08406419** | *-0.08255078* | *-0.236595756* |
| AP2S1 | **0.2827742** | **0.06073913** | *-0.385257867* | *0.449321396* |
| H2AFZ | **0.4905084** | **-0.1456053** | *-0.073837333* | *0.548699747* |
| HIST1H3D | **0.3305871** | **0.01435528** | *0.043848978* | *0.245102389* |
| SNRPD3 | **0.2281217** | **0.120352** | *-0.308635395* | *-0.057536692* |
| HADH | **0.3125671** | **0.04124304** | *0.208387643* | *-0.172145526* |
| MRPL2 | **0.2895643** | **0.06488286** | *0.461187831* | *0.104914175* |
| MRPL21 | **0.2614363** | **0.09356022** | *0.000791283* | *0.223214233* |
| SHMT2 | **0.3097393** | **0.04963072** | *0.474778883* | *-0.389556817* |
| PMPCA | **0.2526976** | **0.1110314** | *0.306644843* | *-0.104539045* |
| MRPS14 | **0.4200725** | **-0.04692106** | *-0.122842671* | *0.010831569* |
| FIS1 | **0.2123813** | **0.16221** | *0.252702547* | *0.132918206* |
| TRAP1 | **0.2682163** | **0.1096949** | *-0.05055777* | *-0.636182998* |
| NDUFB10 | **0.4613524** | **-0.08008794** | *0.029514707* | *-0.081813472* |
| RBM25 | **0.2558964** | **0.125651** | *-0.004185243* | *-0.071945415* |
| REXO2 | **0.213924** | **0.168642** | *-0.689832817* | *-0.540369748* |
| ME2 | **0.2787941** | **0.1070183** | *0.061587695* | *-0.791239502* |
| SLC25A3 | **0.3643021** | **0.02431962** | *-0.201143567* | *-0.180121198* |
| HSD17B8 | **0.1085599** | **0.2821432** | *0.240810713* | *-0.06599413* |
| PHB | **0.3243888** | **0.07038926** | *0.209052953* | *0.483960302* |
| DHX9 | **0.3449158** | **0.05102402** | *0.142044195* | *-0.115914569* |
| MRPL23 | **0.303134** | **0.09491159** | *0.113682997* | *0.152841526* |
| ALDH2 | **0.5469881** | **-0.1472021** | *0.529156643* | *-0.271173778* |
| MRPL11 | **0.3113244** | **0.0908535** | *0.220686233* | *-0.256652706* |
| PTGES2 | **0.3485217** | **0.05380643** | *-0.05084624* | *-0.039977532* |
| RCN2 | **0.290956** | **0.1123665** | *0.205513595* | *-0.223138641* |
| IDH3B | **0.2026283** | **0.2053925** | *-0.028245617* | *-0.087717463* |
| CTNNA1 | **0.3955466** | **0.01292609** | *0.020903177* | *0.380822084* |
| FYN | **0.1278408** | **0.2821432** | *-0.175897557* | *-0.066060311* |
| TMED1 | **0.2944751** | **0.1230039** | *0.12433871* | *0.545802106* |
| LRPPRC | **0.3180183** | **0.1003049** | *-0.02576261* | *-0.30708328* |
| AFG3L2 | **0.3648351** | **0.05380643** | *0.079408637* | *0.076186626* |
| MME | **0.5022684** | **-0.08008794** | *-0.191485249* | *0.172634157* |
| STOML2 | **0.3094164** | **0.1137005** | *0.267876603* | *-0.004150726* |
| MRPL49 | **0.3872502** | **0.03984026** | *0.220237614* | *0.363134984* |
| NDUFS3 | **0.4010966** | **0.02715413** | *0.162074033* | *-0.115298012* |
| NUP93 | **0.3500136** | **0.07997543** | *0.341130923* | *-1.409845766* |
| CPSF1 | **0.4159074** | **0.01578305** | *-0.003623985* | *-0.16357771* |
| PHB2 | **0.3389192** | **0.09626186** | *-0.218531157* | *-0.212985723* |
| MUT | **0.3564801** | **0.07997543** | *0.11201898* | *0.038760058* |
| LONP1 | **0.3430806** | **0.09491159** | *-0.054082497* | *-0.179158158* |
| PCCB | **0.3351533** | **0.102994** | *0.18113237* | *-0.192562928* |
| RRBP1 | **0.4125124** | **0.02573757** | *0.342386403* | *0.435154731* |
| RAB11B | **0.189188** | **0.2533843** | *-0.187612152* | *0.045984378* |
| OGDH | **0.3111781** | **0.1335635** | *0.103691167* | *0.123202425* |
| THOC6 | **0.362895** | **0.08270261** | *0.259885629* | *-0.208730581* |
| ACADVL | **0.3535979** | **0.09220742** | *-0.148945916* | *0.207708348* |
| SDHA | **0.3100539** | **0.1361914** | *0.051752326* | *-0.41760548* |
| DNAJB11 | **0.5606436** | **-0.1124747** | *0.136518373* | *0.529259786* |
| FDXR | **0.3512495** | **0.09895846** | *-0.014893387* | *-1.008481155* |
| BSG | **0.3272398** | **0.1230039** | *0.450824893* | *-0.241543518* |
| CTNNB1 | **0.4347321** | **0.01863419** | *0.032456101* | *-0.262356764* |
| CS | **0.3085061** | **0.1453513** | *-0.186922211* | *-0.059863214* |
| ACO2 | **0.3800912** | **0.08133958** | *-0.182170213* | *0.211381043* |
| CTNND1 | **0.3872628** | **0.0745054** | *-0.113180762* | *-0.167469898* |
| TPR | **0.4692998** | **-0.005782364** | *0.555812677* | *-0.104022269* |
| PNN | **0.3289981** | **0.1348781** | *-0.576756093* | *-0.345980043* |
| SAFB | **0.4579678** | **0.01006364** | *-0.152183503* | *-0.116924939* |
| SEC22B | **0.4444644** | **0.02573757** | *0.146041837* | *0.214756564* |
| MRPS5 | **0.4461362** | **0.02431962** | *-0.002909483* | *-0.335150123* |
| HNRNPL | **0.3730774** | **0.09761087** | *0.37359621* | *-0.293103587* |
| NENF | **0.4213044** | **0.04963072** | *0.239033107* | *-0.214733741* |
| RDH13 | **0.343164** | **0.1296127** | *-0.029169816* | *0.019987548* |
| DDOST | **0.4473839** | **0.02715413** | *0.09496235* | *-0.036825036* |
| MRPL17 | **0.3856204** | **0.09220742** | *0.180926653* | *0.126397041* |
| HIST1H1C | **0.339261** | **0.1401242** | *1.018898517* | *-0.212503775* |
| MRPS28 | **0.3967746** | **0.08542464** | *0.05942467* | *-0.045458604* |
| DLST | **0.2474828** | **0.2375637** | *-0.014034113* | *-0.142667535* |
| ACAD9 | **0.4740027** | **0.01149566** | *0.20308058* | *0.077493174* |
| OCIAD1 | **0.4771267** | **0.008630365** | *-0.06783545* | *-0.160350751* |
| DIABLO | **0.2636235** | **0.2228046** | *-0.434634781* | *0.369342751* |
| HNRNPA2B1 | **0.3688648** | **0.1176951** | *-0.031122563* | *-0.246928781* |
| HTRA2 | **0.163426** | **0.3253864** | *0.092666108* | *-0.211071204* |
| ARL8B | **0.3535041** | **0.1361914** | *-0.294084807* | *0.274317336* |
| AK2 | **0.2560016** | **0.2338881** | *0.235351727* | *0.326922555* |
| MRPL14 | **0.4061303** | **0.08406419** | *-0.001319663* | *0.011534128* |
| HSPA9 | **0.3668539** | **0.125651** | *0.132346457* | *-0.074506982* |
| MRPL39 | **0.3888038** | **0.1043367** | *0.117080165* | *0.02354202* |
| MRPL16 | **0.3397917** | **0.1557492** | *0.170966906* | *-0.342337828* |
| CKMT1B | **0.4467889** | **0.05241581** | *-0.060586394* | *-0.181157571* |
| NDUFS7 | **0.3924105** | **0.1070183** | *-0.076810367* | *0.037883404* |
| ILF2 | **0.4157917** | **0.08678382** | *-0.349865803* | *0.13036035* |
| LMNA | **0.3726634** | **0.1322477** | *0.213479943* | *0.384766632* |
| IMMT | **0.3921623** | **0.1163647** | *-0.09205828* | *-0.495166341* |
| RANBP2 | **0.2509023** | **0.2594232** | *0.035839545* | *0.366447054* |
| HSD17B10 | **0.3354551** | **0.1801479** | *0.067911758* | *0.189060516* |
| GNL3 | **0.3962699** | **0.120352** | *0.281908847* | *0.702025494* |
| ETFB | **0.3390359** | **0.1788739** | *0.865817545* | *-0.290505442* |
| HEXB | **0.2987514** | **0.2203299** | *0.100980647* | *-0.1544505* |
| ACAA1 | **0.2193746** | **0.2998307** | *0.223345477* | *0.696479931* |
| SLC25A10 | **0.4802774** | **0.04124304** | *0.075088163* | *0.110251707* |
| NDUFA13 | **0.432863** | **0.0908535** | *0.554858973* | *0.072317538* |
| SNTB2 | **0.2938446** | **0.230203** | *-0.144855893* | *0.430065353* |
| ABHD11 | **0.362527** | **0.167358** | *-0.380703031* | *0.353873017* |
| HK1 | **0.3088185** | **0.2215678** | *0.13283963* | *0.674216386* |
| HNRNPA1 | **0.3787532** | **0.1660727** | *-0.532322696* | *-0.396651785* |
| BDH1 | **0.3041632** | **0.244887** | *0.045822507* | *0.41521418* |
| HIST1H2AD | **0.4411641** | **0.1137005** | *-0.115390005* | *-0.162417449* |
| MRPS23 | **0.4224309** | **0.1335635** | *-0.093948206* | *0.309921391* |
| ZNF326 | **0.4447003** | **0.1123665** | *0.141494621* | *0.097742872* |
| PGAM5 | **0.4645372** | **0.09356022** | *-0.45666468* | *-0.081977983* |
| ATP1A1 | **0.2802218** | **0.2785796** | *0.463490013* | *0.880123503* |
| AP2B1 | **0.4378872** | **0.1216786** | *0.45205387* | *0.356058272* |
| ATP6V1D | **-0.09922846** | **0.6663021** | *0.08896357* | *0.466971521* |
| VDAC1 | **0.3425356** | **0.2265085** | *-0.05259815* | *-0.110232811* |
| PRPF8 | **0.4517229** | **0.1176951** | *-0.03535594* | *-0.355912232* |
| SLC25A13 | **0.4083353** | **0.16221** | *0.012107787* | *0.736807074* |
| MRPL43 | **0.4434955** | **0.1492593** | *0.584608273* | *0.109786235* |
| AP2A2 | **0.7381359** | **-0.1440104** | *0.180518227* | *0.001163791* |
| ATP2A2 | **0.4489669** | **0.1453513** | *-0.60832656* | *-0.247676297* |
| CDK5RAP3 | **0.2039071** | **0.3961595** | *-0.209360187* | *0.15731927* |
| ETFA | **0.3533496** | **0.2558028** | *-0.224308143* | *-0.242066414* |
| PDHB | **0.3533804** | **0.2570107** | *0.02081006* | *0.048448803* |
| GOT2 | **0.3080055** | **0.3056787** | *0.236388377* | *0.457417524* |
| RANGAP1 | **0.4603715** | **0.1557492** | *-0.049694367* | *0.082801361* |
| IARS2 | **0.2927346** | **0.3276874** | *0.000742427* | *-0.214042641* |
| MDH2 | **0.3389441** | **0.2821432** | *-0.091310273* | *0.009016104* |
| PDHX | **0.3057953** | **0.3173041** | *-0.038364367* | *0.514883052* |
| AARS2 | **0.5018132** | **0.1216786** | *-0.17053121* | *0.117989282* |
| MRPS22 | **0.4717441** | **0.1531568** | *0.278689817* | *-0.046807782* |
| ATP5B | **0.3954522** | **0.230203** | *0.075688227* | *0.059027693* |
| AIFM1 | **0.3972772** | **0.2289726** | *-0.066751033* | *0.314421225* |
| CTSD | **0.3563029** | **0.2714257** | *-0.08322599* | *0.150502493* |
| MRPL18 | **0.4593345** | **0.168642** | *-0.106825517* | *-0.056886053* |
| TSFM | **0.4674116** | **0.1609202** | *0.116469757* | *-0.125368612* |
| NDUFS2 | **0.4957939** | **0.1361914** | *-0.31740775* | *-0.048971813* |
| IVD | **0.3332491** | **0.3021728** | *0.232847408* | *0.203254642* |
| THNSL1 | **0.2454932** | **0.3917678** | *0.140327875* | *-0.50782621* |
| TUFM | **0.3767982** | **0.2606279** | *0.32287524* | *-0.311817454* |
| ATP5A1 | **0.3945677** | **0.244887** | *-0.167954127* | *-0.035600479* |
| ATP5H | **0.4873813** | **0.1531568** | *0.015411027* | *-0.15171876* |
| VDAC2 | **0.3888676** | **0.2533843** | *0.021705463* | *-0.10185411* |
| ECHS1 | **0.3027759** | **0.3402774** | *-0.110650937* | *-0.195629266* |
| ITGB1 | **0.5237973** | **0.1230039** | *-0.43451956* | *0.269466498* |
| ECH1 | **0.3959163** | **0.2509616** | *0.158759997* | *-0.160830271* |
| DECR2 | **0.4229701** | **0.230203** | *0.095453323* | *0.226203946* |
| SMARCC2 | **0.3596205** | **0.2951353** | *-0.154755209* | *-0.507058493* |
| KRT18 | **0.3594426** | **0.2963106** | *-0.64838076* | *2.175515961* |
| NIPSNAP3A | **0.3180825** | **0.3482324** | *1.38074959* | *-2.217516982* |
| SPRYD4 | **0.381259** | **0.2856981** | *0.274741795* | *0.118575547* |
| CTSZ | **0.4935659** | **0.1750454** | *-0.398692143* | *-0.069803329* |
| NSF | **0.3945233** | **0.2750071** | *-0.067794192* | *0.164473345* |
| RAB2A | **0.4303345** | **0.24732** | *0.22476298* | *0.411817079* |
| PRDX3 | **0.2684051** | **0.41792** | *-0.21525073* | *-0.243954071* |
| ACOX1 | **0.5022202** | **0.1852322** | *0.034990924* | *0.406288284* |
| ATP6V0D1 | **0.4098167** | **0.2797684** | *-0.118695203* | *0.419761932* |
| CD9 | **0.4408472** | **0.2521734** | *0.055234578* | *0.408116479* |
| DLD | **0.3383051** | **0.3606452** | *-0.455989337* | *0.058509824* |
| HINT2 | **0.6633043** | **0.0426443** | *0.312632233* | *-0.189115861* |
| OXCT1 | **0.4848332** | **0.2228046** | *0.028417871* | *-0.196307587* |
| PPIF | **0.3562404** | **0.3538878** | *-0.188304142* | *-0.097108359* |
| ZADH2 | **0.3455115** | **0.3718376** | *-0.10819637* | *-0.213489063* |
| HADHA | **0.5336746** | **0.1839629** | *0.105545612* | *-0.041948659* |
| FH | **0.4120455** | **0.3115032** | *0.268287103* | *-0.208820387* |
| SLC25A11 | **0.389954** | **0.3379965** | *0.609371337* | *0.158401996* |
| DECR1 | **0.408418** | **0.3230819** | *0.168994337* | *-0.072345176* |
| GCAT | **0.3352971** | **0.403813** | *0.622964927* | *0.864727553* |
| ATP5O | **0.4718834** | **0.2714257** | *-0.29961841* | *-0.038762311* |
| GSTK1 | **0.5230062** | **0.2351144** | *0.38063353* | *-0.599262064* |
| LETM1 | **0.4572347** | **0.3311319** | *0.071133875* | *0.269307946* |
| MRPS35 | **0.5185731** | **0.2821432** | *-0.62048078* | *0.136092055* |
| SCO1 | **0.5139232** | **0.3021728** | *-0.313467667* | *-0.068206217* |
| UQCRFS1 | **0.369413** | **0.4573317** | *0.154992337* | *-0.027271767* |
| TOMM40 | **0.5846019** | **0.2582174** | *-0.154024633* | *0.206359687* |
| REEP6 | **0.5479621** | **0.3115032** | *0.160557727* | *0.046654038* |
| TOMM70A | **0.3991618** | **0.4719678** | *0.093871597* | *-0.144392454* |
| HMOX1 | **1.130947** | **-0.2567005** | *-0.389612538* | *-0.349288984* |
| HDHD3 | **0.5346913** | **0.3425547** | *0.287956318* | *-0.211331895* |
| HIBADH | **0.4686815** | **0.4135941** | *0.675933207* | *-0.508615669* |
| NDUFA9 | **0.5557462** | **0.3436921** | *0.41113074* | *-0.010882764* |
| UQCRC1 | **0.6081364** | **0.3149864** | *0.35025483* | *0.029696133* |
| ATP5C1 | **0.4858482** | **0.496718** | *0.38670848* | *0.024515749* |
| NAAA | **0.5186067** | **0.5469561** | *0.05668142* | *0.407379162* |
| MRPS25 | **0.5958151** | **0.488515** | *-0.361628747* | *0.064895096* |
| RAB3D | **0.7068381** | **0.444667** | *-0.042056049* | *0.32656114* |
| TOP1 | **0.2522109** | **0.9358369** | *0.076553938* | *-0.101327717* |
| MRPS9 | **0.466748** | **1.100978** | *-0.044396713* | *-0.186481015* |
