# Supplementary material for: A reciprocal feedback between the PDZ binding kinase and androgen receptor drives prostate cancer
Source: Oncogene. 2018 Sep 20;38(7):1136–50. doi: 10.1038/s41388-018-0501-z (PMC6514849; doi:10.1038/s41388-018-0501-z)
Supplement: Supplementary file 7 — Table S2 Warren [file 41388_2018_501_MOESM7_ESM.docx]

|  |  |  |  |  |
| --- | --- | --- | --- | --- |
| Table S2: Core androgen regulated proteins in C4-2 cells | | | | |
|  | **AR knockdown** | **Bicalutamide** | *Bicalutamide* | *R1881* |
| GDF15 | **-1.51837** | **-0.6597226** | *-0.227923533* | *-0.415143733* |
| FKBP5 | **-1.086298** | **-0.3183259** | *-0.573575259* | *4.487376676* |
| RPIA | **-0.544733** | **-0.6896599** | *-0.129842826* | *0.026849106* |
| SAE1 | **-0.5346476** | **-0.6780719** | *-0.198972417* | *-0.289453845* |
| FASN | **-0.6920825** | **-0.3827015** | *-0.255154593* | *1.189846347* |
| PDCD4 | **-0.6948717** | **-0.3566055** | *0.151439297* | *-0.156217041* |
| TOMM34 | **-0.7441587** | **-0.2688168** | *-0.075991833* | *-0.044432032* |
| MAP4 | **-0.6707469** | **-0.293359** | *-0.451778675* | *0.089519211* |
| PBK | **-0.3016705** | **-0.6461121** | *0.714999513* | *-0.271301347* |
| GNL1 | **-0.4900306** | **-0.4208199** | *0.167492656* | *-0.190181759* |
| IMPDH2 | **-0.5742067** | **-0.3346073** | *0.202484497* | *-0.768892077* |
| GCLM | **-0.4687077** | **-0.4111955** | *-0.761741258* | *0.171850685* |
| TCEA1 | **-0.4598725** | **-0.4169624** | *-1.187994147* | *-0.450336352* |
| DTD1 | **-0.3868828** | **-0.479955** | *-0.013786258* | *-0.1082058* |
| GMPPB | **-0.5898894** | **-0.2688168** | *-0.17335801* | *1.389937072* |
| UBE2N | **-0.4971712** | **-0.3382504** | *-0.37986782* | *0.429510239* |
| ABCE1 | **-0.400302** | **-0.4111955** | *0.010357263* | *-0.183699784* |
| VARS | **-0.4928418** | **-0.2986727** | *-0.429716806* | *-0.171805386* |
| CSDE1 | **-0.504839** | **-0.2827896** | *-0.307770517* | *-0.168226787* |
| STMN1 | **-0.389873** | **-0.3808218** | *0.061178891* | *0.174812208* |
| PEF1 | **-0.3531708** | **-0.4111955** | *0.190496266* | *0.140800612* |
| ME1 | **-0.4758185** | **-0.2792838** | *-0.166976673* | *0.164688001* |
| DPP3 | **-0.4330175** | **-0.3057884** | *0.168883076* | *0.109977213* |
| DUT | **-0.4513231** | **-0.2618807** | *0.062706866* | *-0.182631978* |
| TTLL12 | **-0.436585** | **-0.2567005** | *0.018420267* | *0.233120574* |
| IPO4 | **-0.2551408** | **-0.4169624** | *-0.189594453* | *-0.010837325* |
| TARS | **-0.3429762** | **-0.3147326** | *-0.02881558* | *0.372796993* |
| NCAPG | **-0.2971287** | **-0.3529158** | *1.087528423* | *-0.056898982* |
| FXR1 | **-0.3694236** | **-0.2670797** | *-0.107505223* | *-0.32466382* |
| HMGB3 | **-0.2894652** | **-0.2636116** | *0.262836634* | *0.110710029* |
| RANBP2 | **0.2509023** | **0.2594232** | *0.035839545* | *0.366447054* |
| ATP1A1 | **0.2802218** | **0.2785796** | *0.463490013* | *0.880123503* |
| ETFA | **0.3533496** | **0.2558028** | *-0.224308143* | *-0.242066414* |
| PDHB | **0.3533804** | **0.2570107** | *0.02081006* | *0.048448803* |
| GOT2 | **0.3080055** | **0.3056787** | *0.236388377* | *0.457417524* |
| IARS2 | **0.2927346** | **0.3276874** | *0.000742427* | *-0.214042641* |
| MDH2 | **0.3389441** | **0.2821432** | *-0.091310273* | *0.009016104* |
| PDHX | **0.3057953** | **0.3173041** | *-0.038364367* | *0.514883052* |
| CTSD | **0.3563029** | **0.2714257** | *-0.08322599* | *0.150502493* |
| IVD | **0.3332491** | **0.3021728** | *0.232847408* | *0.203254642* |
| TUFM | **0.3767982** | **0.2606279** | *0.32287524* | *-0.311817454* |
| VDAC2 | **0.3888676** | **0.2533843** | *0.021705463* | *-0.10185411* |
| ECHS1 | **0.3027759** | **0.3402774** | *-0.110650937* | *-0.195629266* |
| ECH1 | **0.3959163** | **0.2509616** | *0.158759997* | *-0.160830271* |
| SMARCC2 | **0.3596205** | **0.2951353** | *-0.154755209* | *-0.507058493* |
| KRT18 | **0.3594426** | **0.2963106** | *-0.64838076* | *2.175515961* |
| NIPSNAP3A | **0.3180825** | **0.3482324** | *1.38074959* | *-2.217516982* |
| SPRYD4 | **0.381259** | **0.2856981** | *0.274741795* | *0.118575547* |
| NSF | **0.3945233** | **0.2750071** | *-0.067794192* | *0.164473345* |
| PRDX3 | **0.2684051** | **0.41792** | *-0.21525073* | *-0.243954071* |
| ATP6V0D1 | **0.4098167** | **0.2797684** | *-0.118695203* | *0.419761932* |
| CD9 | **0.4408472** | **0.2521734** | *0.055234578* | *0.408116479* |
| DLD | **0.3383051** | **0.3606452** | *-0.455989337* | *0.058509824* |
| PPIF | **0.3562404** | **0.3538878** | *-0.188304142* | *-0.097108359* |
| ZADH2 | **0.3455115** | **0.3718376** | *-0.10819637* | *-0.213489063* |
| FH | **0.4120455** | **0.3115032** | *0.268287103* | *-0.208820387* |
| SLC25A11 | **0.389954** | **0.3379965** | *0.609371337* | *0.158401996* |
| DECR1 | **0.408418** | **0.3230819** | *0.168994337* | *-0.072345176* |
| GCAT | **0.3352971** | **0.403813** | *0.622964927* | *0.864727553* |
| ATP5O | **0.4718834** | **0.2714257** | *-0.29961841* | *-0.038762311* |
| LETM1 | **0.4572347** | **0.3311319** | *0.071133875* | *0.269307946* |
| MRPS35 | **0.5185731** | **0.2821432** | *-0.62048078* | *0.136092055* |
| SCO1 | **0.5139232** | **0.3021728** | *-0.313467667* | *-0.068206217* |
| UQCRFS1 | **0.369413** | **0.4573317** | *0.154992337* | *-0.027271767* |
| TOMM40 | **0.5846019** | **0.2582174** | *-0.154024633* | *0.206359687* |
| REEP6 | **0.5479621** | **0.3115032** | *0.160557727* | *0.046654038* |
| TOMM70A | **0.3991618** | **0.4719678** | *0.093871597* | *-0.144392454* |
| HDHD3 | **0.5346913** | **0.3425547** | *0.287956318* | *-0.211331895* |
| HIBADH | **0.4686815** | **0.4135941** | *0.675933207* | *-0.508615669* |
| NDUFA9 | **0.5557462** | **0.3436921** | *0.41113074* | *-0.010882764* |
| UQCRC1 | **0.6081364** | **0.3149864** | *0.35025483* | *0.029696133* |
| ATP5C1 | **0.4858482** | **0.496718** | *0.38670848* | *0.024515749* |
| NAAA | **0.5186067** | **0.5469561** | *0.05668142* | *0.407379162* |
| MRPS25 | **0.5958151** | **0.488515** | *-0.361628747* | *0.064895096* |
| RAB3D | **0.7068381** | **0.444667** | *-0.042056049* | *0.32656114* |
| TOP1 | **0.2522109** | **0.9358369** | *0.076553938* | *-0.101327717* |
| MRPS9 | **0.466748** | **1.100978** | *-0.044396713* | *-0.186481015* |
