# Supplementary material for: A reciprocal feedback between the PDZ binding kinase and androgen receptor drives prostate cancer
Source: Oncogene. 2018 Sep 20;38(7):1136–50. doi: 10.1038/s41388-018-0501-z (PMC6514849; doi:10.1038/s41388-018-0501-z)
Supplement: Supplementary file 8 — Table S3 Warren [file 41388_2018_501_MOESM8_ESM.docx]

| Table S3: PBK RIME data showing specific interaction partners of PBK in C4-2 cells (PBK RIME Interactions (IgG filtered) | | | | | | | | | | |
| --- | --- | --- | --- | --- | --- | --- | --- | --- | --- | --- |
|  | | |  |  |  |  |  |  |  |  |
| Accession | Description | Name | Score (Sequest) | _Coverage | _# Unique Peptides | _# Peptides | _# PSMs | # AAs | MW [kDa] | calc. pI |
| Q99798 | Aconitate hydratase, mitochondrial OS=Homo sapiens GN=ACO2 PE=1 SV=2 - [ACON_HUMAN] | ACON | 1.03 | 1.03 | 1 | 1 | 2 | 780 | 85.4 | 7.61 |
| O95573 | Long-chain-fatty-acid--CoA ligase 3 OS=Homo sapiens GN=ACSL3 PE=1 SV=3 - [ACSL3_HUMAN] | ACSL3 | 6.39 | 6.39 | 3 | 3 | 7 | 720 | 80.4 | 8.38 |
| P05141 | ADP/ATP translocase 2 OS=Homo sapiens GN=SLC25A5 PE=1 SV=7 - [ADT2_HUMAN] | ADT2 | 7.38 | 7.38 | 1 | 2 | 10 | 298 | 32.8 | 9.69 |
| P12236 | ADP/ATP translocase 3 OS=Homo sapiens GN=SLC25A6 PE=1 SV=4 - [ADT3_HUMAN] | ADT3 | 11.07 | 11.07 | 2 | 3 | 11 | 298 | 32.8 | 9.74 |
| Q9Y4W6 | AFG3-like protein 2 OS=Homo sapiens GN=AFG3L2 PE=1 SV=2 - [AFG32_HUMAN] | AFG32 | 1 | 1 | 1 | 1 | 1 | 797 | 88.5 | 8.66 |
| Q53H12 | Acylglycerol kinase, mitochondrial OS=Homo sapiens GN=AGK PE=1 SV=2 - [AGK_HUMAN] | AGK | 2.13 | 2.13 | 1 | 1 | 4 | 422 | 47.1 | 8.09 |
| P10275 | Androgen receptor OS=Homo sapiens GN=AR PE=1 SV=2 - [ANDR_HUMAN] | ANDR | 26.66 | 26.66 | 16 | 16 | 70 | 919 | 98.9 | 6.42 |
| P21397 | Amine oxidase [flavin-containing] A OS=Homo sapiens GN=MAOA PE=1 SV=1 - [AOFA_HUMAN] | AOFA | 3.98 | 3.98 | 2 | 2 | 3 | 527 | 59.6 | 7.85 |
| Q9ULZ3 | Apoptosis-associated speck-like protein containing a CARD OS=Homo sapiens GN=PYCARD PE=1 SV=2 - [ASC_HUMAN] | ASC | 4.1 | 4.1 | 1 | 1 | 1 | 195 | 21.6 | 6.34 |
| Q9NVI7 | ATPase family AAA domain-containing protein 3A OS=Homo sapiens GN=ATAD3A PE=1 SV=2 - [ATD3A_HUMAN] | ATD3A | 65.3 | 65.3 | 21 | 48 | 806 | 634 | 71.3 | 8.98 |
| Q5T9A4 | ATPase family AAA domain-containing protein 3B OS=Homo sapiens GN=ATAD3B PE=1 SV=1 - [ATD3B_HUMAN] | ATD3B | 37.04 | 37.04 | 5 | 32 | 469 | 648 | 72.5 | 9.2 |
| P06576 | ATP synthase subunit beta, mitochondrial OS=Homo sapiens GN=ATP5B PE=1 SV=3 - [ATPB_HUMAN] | ATPB | 7.94 | 7.94 | 3 | 3 | 6 | 529 | 56.5 | 5.4 |
| O60242 | Brain-specific angiogenesis inhibitor 3 OS=Homo sapiens GN=BAI3 PE=1 SV=2 - [BAI3_HUMAN] | BAI3 | 1.31 | 1.31 | 1 | 1 | 1 | 1522 | 171.4 | 7.06 |
| Q07021 | Complement component 1 Q subcomponent-binding protein, mitochondrial OS=Homo sapiens GN=C1QBP PE=1 SV=1 - [C1QBP_HUMAN] | C1QBP | 4.96 | 4.96 | 1 | 1 | 2 | 282 | 31.3 | 4.84 |
| P62158 | Calmodulin OS=Homo sapiens GN=CALM1 PE=1 SV=2 - [CALM_HUMAN] | CALM | 10.74 | 10.74 | 1 | 1 | 1 | 149 | 16.8 | 4.22 |
| Q6PK04 | Coiled-coil domain-containing protein 137 OS=Homo sapiens GN=CCDC137 PE=1 SV=1 - [CC137_HUMAN] | CC137 | 3.11 | 3.11 | 1 | 1 | 1 | 289 | 33.2 | 10.93 |
| Q96ER9 | Coiled-coil domain-containing protein 51 OS=Homo sapiens GN=CCDC51 PE=1 SV=2 - [CCD51_HUMAN] | CCD51 | 2.43 | 2.43 | 1 | 1 | 1 | 411 | 45.8 | 8.19 |
| Q4VC31 | Coiled-coil domain-containing protein 58 OS=Homo sapiens GN=CCDC58 PE=1 SV=1 - [CCD58_HUMAN] | CCD58 | 9.03 | 9.03 | 1 | 1 | 2 | 144 | 16.6 | 7.81 |
| Q13740 | CD166 antigen OS=Homo sapiens GN=ALCAM PE=1 SV=2 - [CD166_HUMAN] | CD166 | 3.43 | 3.43 | 1 | 1 | 1 | 583 | 65.1 | 6.25 |
| Q5T1J5 | Putative coiled-coil-helix-coiled-coil-helix domain-containing protein CHCHD2P9, mitochondrial OS=Homo sapiens GN=CHCHD2P9 PE=5 SV=1 - [CHCH9_HUMAN] | CHCH9 | 18.54 | 18.54 | 1 | 1 | 4 | 151 | 15.5 | 9.89 |
| O75390 | Citrate synthase, mitochondrial OS=Homo sapiens GN=CS PE=1 SV=2 - [CISY_HUMAN] | CISY | 1.93 | 1.93 | 1 | 1 | 4 | 466 | 51.7 | 8.32 |
| Q9H078 | Caseinolytic peptidase B protein homolog OS=Homo sapiens GN=CLPB PE=1 SV=1 - [CLPB_HUMAN] | CLPB | 19.8 | 19.8 | 11 | 11 | 41 | 707 | 78.7 | 9.01 |
| Q14028 | Cyclic nucleotide-gated cation channel beta-1 OS=Homo sapiens GN=CNGB1 PE=1 SV=2 - [CNGB1_HUMAN] | CNGB1 | 0.48 | 0.48 | 1 | 1 | 1 | 1251 | 139.6 | 4.81 |
| Q9NYJ1 | Cytochrome c oxidase assembly factor 4 homolog, mitochondrial OS=Homo sapiens GN=COA4 PE=1 SV=2 - [COA4_HUMAN] | COA4 | 19.54 | 19.54 | 1 | 1 | 2 | 87 | 10.1 | 6.04 |
| Q96BR5 | Cytochrome c oxidase assembly factor 7 OS=Homo sapiens GN=COA7 PE=1 SV=2 - [COA7_HUMAN] | COA7 | 3.9 | 3.9 | 1 | 1 | 4 | 231 | 25.7 | 6.02 |
| Q14061 | Cytochrome c oxidase copper chaperone OS=Homo sapiens GN=COX17 PE=1 SV=2 - [COX17_HUMAN] | COX17 |  | 25.4 | 1 | 1 | 2 | 63 | 6.9 | 7.24 |
| P00403 | Cytochrome c oxidase subunit 2 OS=Homo sapiens GN=MT-CO2 PE=1 SV=1 - [COX2_HUMAN] | COX2 | 7.49 | 7.49 | 2 | 2 | 3 | 227 | 25.5 | 4.82 |
| P13073 | Cytochrome c oxidase subunit 4 isoform 1, mitochondrial OS=Homo sapiens GN=COX4I1 PE=1 SV=1 - [COX41_HUMAN] | COX41 | 12.43 | 12.43 | 2 | 2 | 5 | 169 | 19.6 | 9.51 |
| P09669 | Cytochrome c oxidase subunit 6C OS=Homo sapiens GN=COX6C PE=1 SV=2 - [COX6C_HUMAN] | COX6C | 10.67 | 10.67 | 1 | 1 | 1 | 75 | 8.8 | 10.39 |
| Q86YQ8 | Copine-8 OS=Homo sapiens GN=CPNE8 PE=1 SV=2 - [CPNE8_HUMAN] | CPNE8 | 1.6 | 1.6 | 1 | 1 | 2 | 564 | 63.1 | 5.96 |
| Q9HB71 | Calcyclin-binding protein OS=Homo sapiens GN=CACYBP PE=1 SV=2 - [CYBP_HUMAN] | CYBP | 3.07 | 3.07 | 1 | 1 | 2 | 228 | 26.2 | 8.25 |
| Q9NR28 | Diablo homolog, mitochondrial OS=Homo sapiens GN=DIABLO PE=1 SV=1 - [DBLOH_HUMAN] | DBLOH | 13.81 | 13.81 | 2 | 2 | 2 | 239 | 27.1 | 5.9 |
| O15523 | ATP-dependent RNA helicase DDX3Y OS=Homo sapiens GN=DDX3Y PE=1 SV=2 - [DDX3Y_HUMAN] | DDX3Y | 3.18 | 3.18 | 1 | 2 | 6 | 660 | 73.1 | 7.55 |
| Q86TM3 | Probable ATP-dependent RNA helicase DDX53 OS=Homo sapiens GN=DDX53 PE=1 SV=3 - [DDX53_HUMAN] | DDX53 | 1.9 | 1.9 | 1 | 1 | 2 | 631 | 71.1 | 9.07 |
| Q7Z478 | ATP-dependent RNA helicase DHX29 OS=Homo sapiens GN=DHX29 PE=1 SV=2 - [DHX29_HUMAN] | DHX29 | 0.66 | 0.66 | 1 | 1 | 3 | 1369 | 155.1 | 8.09 |
| Q7L2E3 | Putative ATP-dependent RNA helicase DHX30 OS=Homo sapiens GN=DHX30 PE=1 SV=1 - [DHX30_HUMAN] | DHX30 | 2.18 | 2.18 | 2 | 2 | 5 | 1194 | 133.9 | 8.78 |
| O00148 | ATP-dependent RNA helicase DDX39A OS=Homo sapiens GN=DDX39A PE=1 SV=2 - [DX39A_HUMAN] | DX39A | 2.34 | 2.34 | 1 | 1 | 2 | 427 | 49.1 | 5.68 |
| Q8TD57 | Dynein heavy chain 3, axonemal OS=Homo sapiens GN=DNAH3 PE=2 SV=1 - [DYH3_HUMAN] | DYH3 | 0.46 | 0.46 | 1 | 1 | 1 | 4116 | 470.5 | 6.43 |
| Q9HAF1 | Chromatin modification-related protein MEAF6 OS=Homo sapiens GN=MEAF6 PE=1 SV=1 - [EAF6_HUMAN] | EAF6 | 20.94 | 20.94 | 1 | 1 | 4 | 191 | 21.6 | 9.32 |
| Q13011 | Delta(3,5)-Delta(2,4)-dienoyl-CoA isomerase, mitochondrial OS=Homo sapiens GN=ECH1 PE=1 SV=2 - [ECH1_HUMAN] | ECH1 | 6.71 | 6.71 | 1 | 1 | 1 | 328 | 35.8 | 8 |
| P49411 | Elongation factor Tu, mitochondrial OS=Homo sapiens GN=TUFM PE=1 SV=2 - [EFTU_HUMAN] | EFTU | 5.75 | 5.75 | 2 | 2 | 7 | 452 | 49.5 | 7.61 |
| Q13347 | Eukaryotic translation initiation factor 3 subunit I OS=Homo sapiens GN=EIF3I PE=1 SV=1 - [EIF3I_HUMAN] | EIF3I | 4.92 | 4.92 | 1 | 1 | 1 | 325 | 36.5 | 5.64 |
| Q9Y262 | Eukaryotic translation initiation factor 3 subunit L OS=Homo sapiens GN=EIF3L PE=1 SV=1 - [EIF3L_HUMAN] | EIF3L | 1.42 | 1.42 | 1 | 1 | 1 | 564 | 66.7 | 6.34 |
| Q9NY74 | Ewing's tumor-associated antigen 1 OS=Homo sapiens GN=ETAA1 PE=1 SV=2 - [ETAA1_HUMAN] | ETAA1 | 0.86 | 0.86 | 1 | 1 | 1 | 926 | 103.4 | 7.62 |
| P13804 | Electron transfer flavoprotein subunit alpha, mitochondrial OS=Homo sapiens GN=ETFA PE=1 SV=1 - [ETFA_HUMAN] | ETFA | 3.9 | 3.9 | 1 | 1 | 2 | 333 | 35.1 | 8.38 |
| P38117 | Electron transfer flavoprotein subunit beta OS=Homo sapiens GN=ETFB PE=1 SV=3 - [ETFB_HUMAN] | ETFB | 4.71 | 4.71 | 1 | 1 | 1 | 255 | 27.8 | 8.1 |
| Q96C01 | Protein FAM136A OS=Homo sapiens GN=FAM136A PE=1 SV=1 - [F136A_HUMAN] | F136A | 12.32 | 12.32 | 2 | 2 | 5 | 138 | 15.6 | 7.61 |
| Q96A26 | Protein FAM162A OS=Homo sapiens GN=FAM162A PE=1 SV=2 - [F162A_HUMAN] | F162A | 7.14 | 7.14 | 1 | 1 | 2 | 154 | 17.3 | 9.77 |
| Q6PEV8 | Protein FAM199X OS=Homo sapiens GN=FAM199X PE=1 SV=1 - [F199X_HUMAN] | F199X | 6.19 | 6.19 | 1 | 1 | 1 | 388 | 42.8 | 5.08 |
| P02751 | Fibronectin OS=Homo sapiens GN=FN1 PE=1 SV=4 - [FINC_HUMAN] | FINC | 0.59 | 0.59 | 1 | 1 | 4 | 2386 | 262.5 | 5.71 |
| Q13451 | Peptidyl-prolyl cis-trans isomerase FKBP5 OS=Homo sapiens GN=FKBP5 PE=1 SV=2 - [FKBP5_HUMAN] | FKBP5 | 3.94 | 3.94 | 1 | 1 | 1 | 457 | 51.2 | 5.9 |
| P07954 | Fumarate hydratase, mitochondrial OS=Homo sapiens GN=FH PE=1 SV=3 - [FUMH_HUMAN] | FUMH | 2.75 | 2.75 | 1 | 1 | 1 | 510 | 54.6 | 8.76 |
| Q495W5 | Alpha-(1,3)-fucosyltransferase 11 OS=Homo sapiens GN=FUT11 PE=1 SV=1 - [FUT11_HUMAN] | FUT11 | 4.07 | 4.07 | 1 | 1 | 1 | 492 | 55.8 | 5.94 |
| Q9HD26 | Golgi-associated PDZ and coiled-coil motif-containing protein OS=Homo sapiens GN=GOPC PE=1 SV=1 - [GOPC_HUMAN] | GOPC | 1.95 | 1.95 | 1 | 1 | 1 | 462 | 50.5 | 5.92 |
| Q96QV6 | Histone H2A type 1-A OS=Homo sapiens GN=HIST1H2AA PE=1 SV=3 - [H2A1A_HUMAN] | H2A1A | 29.77 | 29.77 | 1 | 3 | 12 | 131 | 14.2 | 10.86 |
| Q16777 | Histone H2A type 2-C OS=Homo sapiens GN=HIST2H2AC PE=1 SV=4 - [H2A2C_HUMAN] | H2A2C | 58.14 | 58.14 | 3 | 5 | 27 | 129 | 14 | 10.9 |
| O00165 | HCLS1-associated protein X-1 OS=Homo sapiens GN=HAX1 PE=1 SV=2 - [HAX1_HUMAN] | HAX1 | 37.28 | 37.28 | 7 | 7 | 19 | 279 | 31.6 | 4.92 |
| P17066 | Heat shock 70 kDa protein 6 OS=Homo sapiens GN=HSPA6 PE=1 SV=2 - [HSP76_HUMAN] | HSP76 | 7.62 | 7.62 | 1 | 4 | 18 | 643 | 71 | 6.14 |
| P04792 | Heat shock protein beta-1 OS=Homo sapiens GN=HSPB1 PE=1 SV=2 - [HSPB1_HUMAN] | HSPB1 | 17.07 | 17.07 | 3 | 3 | 8 | 205 | 22.8 | 6.4 |
| O75874 | Isocitrate dehydrogenase [NADP] cytoplasmic OS=Homo sapiens GN=IDH1 PE=1 SV=2 - [IDHC_HUMAN] | IDHC | 2.66 | 2.66 | 1 | 1 | 1 | 414 | 46.6 | 7.01 |
| P41091 | Eukaryotic translation initiation factor 2 subunit 3 OS=Homo sapiens GN=EIF2S3 PE=1 SV=3 - [IF2G_HUMAN] | IF2G | 2.97 | 2.97 | 1 | 1 | 1 | 472 | 51.1 | 8.4 |
| P63241 | Eukaryotic translation initiation factor 5A-1 OS=Homo sapiens GN=EIF5A PE=1 SV=2 - [IF5A1_HUMAN] | IF5A1 | 25.97 | 25.97 | 2 | 2 | 3 | 154 | 16.8 | 5.24 |
| Q16891 | Mitochondrial inner membrane protein OS=Homo sapiens GN=IMMT PE=1 SV=1 - [IMMT_HUMAN] | IMMT | 2.64 | 2.64 | 2 | 2 | 2 | 758 | 83.6 | 6.48 |
| P04259 | Keratin, type II cytoskeletal 6B OS=Homo sapiens GN=KRT6B PE=1 SV=5 - [K2C6B_HUMAN] | K2C6B | 22.52 | 22.52 | 1 | 12 | 39 | 564 | 60 | 8 |
| P54819 | Adenylate kinase 2, mitochondrial OS=Homo sapiens GN=AK2 PE=1 SV=2 - [KAD2_HUMAN] | KAD2 | 21.76 | 27.2 | 4 | 4 | 14 | 239 | 26.5 | 7.81 |
| P12532 | Creatine kinase U-type, mitochondrial OS=Homo sapiens GN=CKMT1A PE=1 SV=1 - [KCRU_HUMAN] | KCRU | 16.79 | 16.79 | 5 | 5 | 24 | 417 | 47 | 8.34 |
| Q3LHN1 | Keratin-associated protein 21-3 OS=Homo sapiens GN=KRTAP21-3 PE=3 SV=1 - [KR213_HUMAN] | KR213 | 10.34 | 10.34 | 1 | 1 | 3 | 58 | 6.5 | 7.56 |
| Q9BQ66 | Keratin-associated protein 4-12 OS=Homo sapiens GN=KRTAP4-12 PE=1 SV=1 - [KR412_HUMAN] | KR412 | 39.8 | 39.8 | 1 | 1 | 4 | 201 | 21.4 | 7.88 |
| Q02252 | Methylmalonate-semialdehyde dehydrogenase [acylating], mitochondrial OS=Homo sapiens GN=ALDH6A1 PE=1 SV=2 - [MMSA_HUMAN] | MMSA | 1.87 | 1.87 | 1 | 1 | 2 | 535 | 57.8 | 8.5 |
| Q00325 | Phosphate carrier protein, mitochondrial OS=Homo sapiens GN=SLC25A3 PE=1 SV=2 - [MPCP_HUMAN] | MPCP | 3.31 | 3.31 | 1 | 1 | 4 | 362 | 40.1 | 9.38 |
| Q9Y6C9 | Mitochondrial carrier homolog 2 OS=Homo sapiens GN=MTCH2 PE=1 SV=1 - [MTCH2_HUMAN] | MTCH2 | 9.9 | 9.9 | 2 | 2 | 6 | 303 | 33.3 | 7.97 |
| P60660 | Myosin light polypeptide 6 OS=Homo sapiens GN=MYL6 PE=1 SV=2 - [MYL6_HUMAN] | MYL6 | 10.6 | 10.6 | 1 | 1 | 3 | 151 | 16.9 | 4.65 |
| P22392 | Nucleoside diphosphate kinase B OS=Homo sapiens GN=NME2 PE=1 SV=1 - [NDKB_HUMAN] | NDKB | 19.08 | 19.08 | 2 | 2 | 6 | 152 | 17.3 | 8.41 |
| O00483 | NADH dehydrogenase [ubiquinone] 1 alpha subcomplex subunit 4 OS=Homo sapiens GN=NDUFA4 PE=1 SV=1 - [NDUA4_HUMAN] | NDUA4 | 9.88 | 9.88 | 1 | 1 | 3 | 81 | 9.4 | 9.38 |
| O95139 | NADH dehydrogenase [ubiquinone] 1 beta subcomplex subunit 6 OS=Homo sapiens GN=NDUFB6 PE=1 SV=3 - [NDUB6_HUMAN] | NDUB6 | 7.81 | 7.81 | 1 | 1 | 3 | 128 | 15.5 | 9.63 |
| O96000 | NADH dehydrogenase [ubiquinone] 1 beta subcomplex subunit 10 OS=Homo sapiens GN=NDUFB10 PE=1 SV=3 - [NDUBA_HUMAN] | NDUBA | 8.14 | 8.14 | 1 | 1 | 6 | 172 | 20.8 | 8.48 |
| A6NND4 | Olfactory receptor 2AT4 OS=Homo sapiens GN=OR2AT4 PE=2 SV=1 - [O2AT4_HUMAN] | O2AT4 | 12.5 | 12.5 | 1 | 1 | 1 | 320 | 35.5 | 6.7 |
| Q02218 | 2-oxoglutarate dehydrogenase, mitochondrial OS=Homo sapiens GN=OGDH PE=1 SV=3 - [ODO1_HUMAN] | ODO1 | 1.17 | 1.17 | 1 | 1 | 2 | 1023 | 115.9 | 6.86 |
| O60313 | Dynamin-like 120 kDa protein, mitochondrial OS=Homo sapiens GN=OPA1 PE=1 SV=3 - [OPA1_HUMAN] | OPA1 | 20.31 | 20.31 | 16 | 16 | 45 | 960 | 111.6 | 7.87 |
| Q96DU9 | Polyadenylate-binding protein 5 OS=Homo sapiens GN=PABPC5 PE=2 SV=1 - [PABP5_HUMAN] | PABP5 | 2.36 | 2.36 | 1 | 1 | 1 | 382 | 43.3 | 9.51 |
| P09874 | Poly [ADP-ribose] polymerase 1 OS=Homo sapiens GN=PARP1 PE=1 SV=4 - [PARP1_HUMAN] | PARP1 | 0 | 0.99 | 1 | 1 | 1 | 1014 | 113 | 8.88 |
| Q96HS1 | Serine/threonine-protein phosphatase PGAM5, mitochondrial OS=Homo sapiens GN=PGAM5 PE=1 SV=2 - [PGAM5_HUMAN] | PGAM5 | 3.46 | 3.46 | 1 | 1 | 2 | 289 | 32 | 8.68 |
| P00558 | Phosphoglycerate kinase 1 OS=Homo sapiens GN=PGK1 PE=1 SV=3 - [PGK1_HUMAN] | PGK1 | 10.07 | 10.07 | 3 | 3 | 7 | 417 | 44.6 | 8.1 |
| Q13018 | Secretory phospholipase A2 receptor OS=Homo sapiens GN=PLA2R1 PE=1 SV=2 - [PLA2R_HUMAN] | PLA2R | 2.73 | 2.73 | 1 | 1 | 1 | 1463 | 168.5 | 6.11 |
| Q9HBL7 | Plasminogen receptor (KT) OS=Homo sapiens GN=PLGRKT PE=1 SV=1 - [PLRKT_HUMAN] | PLRKT | 21.09 | 21.09 | 3 | 3 | 23 | 147 | 17.2 | 9.58 |
| P30048 | Thioredoxin-dependent peroxide reductase, mitochondrial OS=Homo sapiens GN=PRDX3 PE=1 SV=3 - [PRDX3_HUMAN] | PRDX3 | 4.3 | 4.3 | 1 | 1 | 2 | 256 | 27.7 | 7.78 |
| P28066 | Proteasome subunit alpha type-5 OS=Homo sapiens GN=PSMA5 PE=1 SV=3 - [PSA5_HUMAN] | PSA5 | 7.88 | 7.88 | 1 | 1 | 1 | 241 | 26.4 | 4.79 |
| P61026 | Ras-related protein Rab-10 OS=Homo sapiens GN=RAB10 PE=1 SV=1 - [RAB10_HUMAN] | RAB10 | 5.5 | 5.5 | 1 | 1 | 4 | 200 | 22.5 | 8.38 |
| Q9UKM9 | RNA-binding protein Raly OS=Homo sapiens GN=RALY PE=1 SV=1 - [RALY_HUMAN] | RALY | 2.94 | 2.94 | 1 | 1 | 4 | 306 | 32.4 | 9.17 |
| Q09028 | Histone-binding protein RBBP4 OS=Homo sapiens GN=RBBP4 PE=1 SV=3 - [RBBP4_HUMAN] | RBBP4 | 3.76 | 3.76 | 2 | 2 | 2 | 425 | 47.6 | 4.89 |
| P38159 | RNA-binding motif protein, X chromosome OS=Homo sapiens GN=RBMX PE=1 SV=3 - [RBMX_HUMAN] | RBMX | 11.51 | 11.51 | 5 | 5 | 17 | 391 | 42.3 | 10.05 |
| Q9UNA1 | Rho GTPase-activating protein 26 OS=Homo sapiens GN=ARHGAP26 PE=1 SV=1 - [RHG26_HUMAN] | RHG26 | 3.56 | 3.56 | 1 | 1 | 1 | 814 | 92.2 | 6.64 |
| P27635 | 60S ribosomal protein L10 OS=Homo sapiens GN=RPL10 PE=1 SV=4 - [RL10_HUMAN] | RL10 | 5.14 | 5.14 | 1 | 1 | 1 | 214 | 24.6 | 10.08 |
| P40429 | 60S ribosomal protein L13a OS=Homo sapiens GN=RPL13A PE=1 SV=2 - [RL13A_HUMAN] | RL13A | 16.26 | 16.26 | 3 | 3 | 8 | 203 | 23.6 | 10.93 |
| Q02543 | 60S ribosomal protein L18a OS=Homo sapiens GN=RPL18A PE=1 SV=2 - [RL18A_HUMAN] | RL18A | 3.98 | 3.98 | 1 | 1 | 1 | 176 | 20.7 | 10.71 |
| P62891 | 60S ribosomal protein L39 OS=Homo sapiens GN=RPL39 PE=1 SV=2 - [RL39_HUMAN] | RL39 | 19.61 | 19.61 | 1 | 1 | 1 | 51 | 6.4 | 12.56 |
| P32969 | 60S ribosomal protein L9 OS=Homo sapiens GN=RPL9 PE=1 SV=1 - [RL9_HUMAN] | RL9 | 7.29 | 7.29 | 1 | 1 | 4 | 192 | 21.8 | 9.95 |
| Q9NZE8 | 39S ribosomal protein L35, mitochondrial OS=Homo sapiens GN=MRPL35 PE=1 SV=3 - [RM35_HUMAN] | RM35 | 5.32 | 5.32 | 1 | 1 | 1 | 188 | 21.5 | 11.3 |
| Q96DB5 | Regulator of microtubule dynamics protein 1 OS=Homo sapiens GN=RMDN1 PE=1 SV=1 - [RMD1_HUMAN] | RMD1 | 6.37 | 6.37 | 2 | 2 | 8 | 314 | 35.8 | 8.5 |
| P04843 | Dolichyl-diphosphooligosaccharide--protein glycosyltransferase subunit 1 OS=Homo sapiens GN=RPN1 PE=1 SV=1 - [RPN1_HUMAN] | RPN1 | 2.8 | 2.8 | 1 | 1 | 4 | 607 | 68.5 | 6.38 |
| Q15424 | Scaffold attachment factor B1 OS=Homo sapiens GN=SAFB PE=1 SV=4 - [SAFB1_HUMAN] | SAFB1 | 0.98 | 0.98 | 1 | 1 | 4 | 915 | 102.6 | 5.47 |
| O75880 | Protein SCO1 homolog, mitochondrial OS=Homo sapiens GN=SCO1 PE=1 SV=1 - [SCO1_HUMAN] | SCO1 | 17.28 | 17.28 | 3 | 3 | 9 | 301 | 33.8 | 8.88 |
| O43819 | Protein SCO2 homolog, mitochondrial OS=Homo sapiens GN=SCO2 PE=1 SV=3 - [SCO2_HUMAN] | SCO2 | 19.55 | 26.32 | 3 | 3 | 7 | 266 | 29.8 | 8.85 |
| P31040 | Succinate dehydrogenase [ubiquinone] flavoprotein subunit, mitochondrial OS=Homo sapiens GN=SDHA PE=1 SV=2 - [SDHA_HUMAN] | SDHA | 4.07 | 4.07 | 2 | 2 | 6 | 664 | 72.6 | 7.39 |
| Q969G3 | SWI/SNF-related matrix-associated actin-dependent regulator of chromatin subfamily E member 1 OS=Homo sapiens GN=SMARCE1 PE=1 SV=2 - [SMCE1_HUMAN] | SMCE1 | 6.08 | 6.08 | 2 | 2 | 4 | 411 | 46.6 | 4.88 |
| Q13243 | Serine/arginine-rich splicing factor 5 OS=Homo sapiens GN=SRSF5 PE=1 SV=1 - [SRSF5_HUMAN] | SRSF5 | 3.31 | 3.31 | 1 | 1 | 4 | 272 | 31.2 | 11.59 |
| P28290 | Sperm-specific antigen 2 OS=Homo sapiens GN=SSFA2 PE=1 SV=3 - [SSFA2_HUMAN] | SSFA2 | 0.87 | 0.87 | 1 | 1 | 2 | 1259 | 138.3 | 5.19 |
| Q9UJZ1 | Stomatin-like protein 2, mitochondrial OS=Homo sapiens GN=STOML2 PE=1 SV=1 - [STML2_HUMAN] | STML2 | 15.45 | 15.45 | 3 | 3 | 5 | 356 | 38.5 | 7.39 |
| Q9UH99 | SUN domain-containing protein 2 OS=Homo sapiens GN=SUN2 PE=1 SV=3 - [SUN2_HUMAN] | SUN2 | 2.93 | 2.93 | 1 | 1 | 1 | 717 | 80.3 | 6.73 |
| Q9Y285 | Phenylalanine--tRNA ligase alpha subunit OS=Homo sapiens GN=FARSA PE=1 SV=3 - [SYFA_HUMAN] | SYFA | 1.38 | 1.38 | 1 | 1 | 1 | 508 | 57.5 | 7.8 |
| Q9NSE4 | Isoleucine--tRNA ligase, mitochondrial OS=Homo sapiens GN=IARS2 PE=1 SV=2 - [SYIM_HUMAN] | SYIM | 1.68 | 1.68 | 1 | 1 | 1 | 1012 | 113.7 | 7.2 |
| P37802 | Transgelin-2 OS=Homo sapiens GN=TAGLN2 PE=1 SV=3 - [TAGL2_HUMAN] | TAGL2 | 9.05 | 9.05 | 1 | 1 | 1 | 199 | 22.4 | 8.25 |
| P68371 | Tubulin beta-4B chain OS=Homo sapiens GN=TUBB4B PE=1 SV=1 - [TBB4B_HUMAN] | TBB4B | 37.53 | 37.53 | 3 | 13 | 90 | 445 | 49.8 | 4.89 |
| P07437 | Tubulin beta chain OS=Homo sapiens GN=TUBB PE=1 SV=2 - [TBB5_HUMAN] | TBB5 | 45.05 | 45.05 | 6 | 16 | 104 | 444 | 49.6 | 4.89 |
| P17987 | T-complex protein 1 subunit alpha OS=Homo sapiens GN=TCP1 PE=1 SV=1 - [TCPA_HUMAN] | TCPA | 1.8 | 1.8 | 1 | 1 | 1 | 556 | 60.3 | 6.11 |
| P49368 | T-complex protein 1 subunit gamma OS=Homo sapiens GN=CCT3 PE=1 SV=4 - [TCPG_HUMAN] | TCPG | 2.02 | 2.02 | 1 | 1 | 2 | 545 | 60.5 | 6.49 |
| Q99832 | T-complex protein 1 subunit eta OS=Homo sapiens GN=CCT7 PE=1 SV=2 - [TCPH_HUMAN] | TCPH | 4.05 | 4.05 | 1 | 1 | 1 | 543 | 59.3 | 7.65 |
| P24752 | Acetyl-CoA acetyltransferase, mitochondrial OS=Homo sapiens GN=ACAT1 PE=1 SV=1 - [THIL_HUMAN] | THIL | 7.26 | 7.26 | 2 | 2 | 4 | 427 | 45.2 | 8.85 |
| Q13263 | Transcription intermediary factor 1-beta OS=Homo sapiens GN=TRIM28 PE=1 SV=5 - [TIF1B_HUMAN] | TIF1B | 7.31 | 7.31 | 5 | 5 | 10 | 835 | 88.5 | 5.77 |
| Q9BVV7 | Mitochondrial import inner membrane translocase subunit Tim21 OS=Homo sapiens GN=TIMM21 PE=1 SV=1 - [TIM21_HUMAN] | TIM21 | 17.34 | 17.34 | 4 | 4 | 10 | 248 | 28.2 | 9.7 |
| O14925 | Mitochondrial import inner membrane translocase subunit Tim23 OS=Homo sapiens GN=TIMM23 PE=1 SV=1 - [TIM23_HUMAN] | TIM23 | 19.62 | 19.62 | 1 | 1 | 1 | 209 | 21.9 | 8.6 |
| O43615 | Mitochondrial import inner membrane translocase subunit TIM44 OS=Homo sapiens GN=TIMM44 PE=1 SV=2 - [TIM44_HUMAN] | TIM44 | 12.61 | 12.61 | 5 | 5 | 17 | 452 | 51.3 | 8.32 |
| Q3ZCQ8 | Mitochondrial import inner membrane translocase subunit TIM50 OS=Homo sapiens GN=TIMM50 PE=1 SV=2 - [TIM50_HUMAN] | TIM50 | 12.46 | 12.46 | 3 | 3 | 12 | 353 | 39.6 | 8.37 |
| P29401 | Transketolase OS=Homo sapiens GN=TKT PE=1 SV=3 - [TKT_HUMAN] | TKT | 2.89 | 2.89 | 1 | 1 | 1 | 623 | 67.8 | 7.66 |
| Q9NYK1 | Toll-like receptor 7 OS=Homo sapiens GN=TLR7 PE=1 SV=1 - [TLR7_HUMAN] | TLR7 | 2.29 | 2.29 | 1 | 2 | 3 | 1049 | 120.8 | 8.21 |
| Q15388 | Mitochondrial import receptor subunit TOM20 homolog OS=Homo sapiens GN=TOMM20 PE=1 SV=1 - [TOM20_HUMAN] | TOM20 | 8.97 | 8.97 | 1 | 1 | 4 | 145 | 16.3 | 8.6 |
| Q9NS69 | Mitochondrial import receptor subunit TOM22 homolog OS=Homo sapiens GN=TOMM22 PE=1 SV=3 - [TOM22_HUMAN] | TOM22 | 25.35 | 25.35 | 2 | 2 | 6 | 142 | 15.5 | 4.34 |
| O96008 | Mitochondrial import receptor subunit TOM40 homolog OS=Homo sapiens GN=TOMM40 PE=1 SV=1 - [TOM40_HUMAN] | TOM40 | 14.96 | 14.96 | 4 | 4 | 11 | 361 | 37.9 | 7.25 |
| Q96KB5 | Lymphokine-activated killer T-cell-originated protein kinase OS=Homo sapiens GN=PBK PE=1 SV=3 - [TOPK_HUMAN] | TOPK | 17.08 | 17.08 | 5 | 5 | 13 | 322 | 36.1 | 5.12 |
| Q9Y2W1 | Thyroid hormone receptor-associated protein 3 OS=Homo sapiens GN=THRAP3 PE=1 SV=2 - [TR150_HUMAN] | TR150 | 1.88 | 1.88 | 1 | 1 | 2 | 955 | 108.6 | 10.15 |
| Q13595 | Transformer-2 protein homolog alpha OS=Homo sapiens GN=TRA2A PE=1 SV=1 - [TRA2A_HUMAN] | TRA2A | 7.09 | 7.09 | 2 | 2 | 4 | 282 | 32.7 | 11.27 |
| P62995 | Transformer-2 protein homolog beta OS=Homo sapiens GN=TRA2B PE=1 SV=1 - [TRA2B_HUMAN] | TRA2B | 5.9 | 5.9 | 2 | 2 | 2 | 288 | 33.6 | 11.25 |
| P45880 | Voltage-dependent anion-selective channel protein 2 OS=Homo sapiens GN=VDAC2 PE=1 SV=2 - [VDAC2_HUMAN] | VDAC2 | 19.73 | 19.73 | 5 | 5 | 12 | 294 | 31.5 | 7.56 |
| P13010 | X-ray repair cross-complementing protein 5 OS=Homo sapiens GN=XRCC5 PE=1 SV=3 - [XRCC5_HUMAN] | XRCC5 | 2.19 | 2.19 | 1 | 1 | 2 | 732 | 82.7 | 5.81 |
| Q96TA2 | ATP-dependent zinc metalloprotease YME1L1 OS=Homo sapiens GN=YME1L1 PE=1 SV=2 - [YMEL1_HUMAN] | YMEL1 | 1.68 | 1.68 | 1 | 1 | 1 | 773 | 86.4 | 8.76 |
